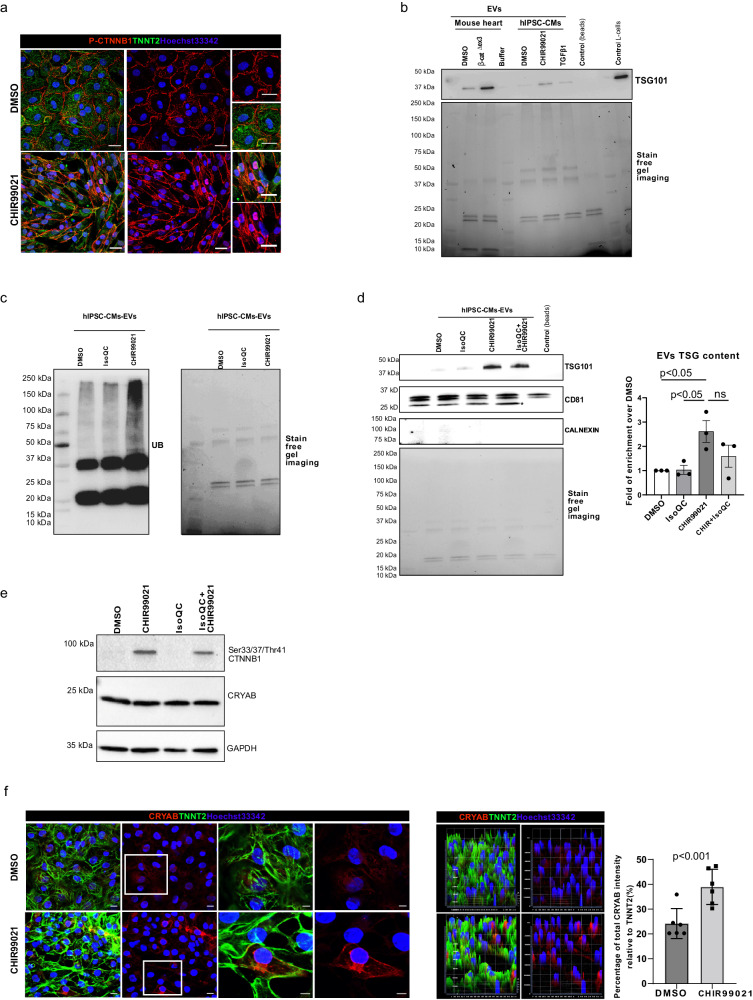# Author Correction: Single-cell transcriptomics reveal extracellular vesicles secretion with a cardiomyocyte proteostasis signature during pathological remodeling

**DOI:** 10.1038/s42003-024-06319-x

**Published:** 2024-05-23

**Authors:** Eric Schoger, Federico Bleckwedel, Giulia Germena, Cheila Rocha, Petra Tucholla, Izzatullo Sobitov, Wiebke Möbius, Maren Sitte, Christof Lenz, Mostafa Samak, Rabea Hinkel, Zoltán V. Varga, Zoltán Giricz, Gabriela Salinas, Julia C. Gross, Laura C. Zelarayán

**Affiliations:** 1 Institute of Pharmacology and Toxicology, University Medical Center Göttingen (UMG), 37075 Göttingen, Germany; 2 German Center for Cardiovascular Research (DZHK) partner site Göttingen, 37075 Göttingen, Germany; 3 Cluster of Excellence “Multiscale Bioimaging: from Molecular Machines to Networks of Excitable Cells” (MBExC), University of Göttingen, 37075 Göttingen, Germany; 4 Laboratory Animal Science Unit, Leibnitz-Institut für Primatenforschung, Deutsches Primatenzentrum GmbH, 37075 Göttingen, Germany; 5 Max-Planck-Institute for Multidisciplinary Sciences, 37075 Göttingen, Germany; 6 NGS Integrative Genomics Core Unit (NIG), University Medical Center Göttingen (UMG), 37075 Göttingen, Germany; 7 Department of Clinical Chemistry, University Medical Center Göttingen (UMG), 37075 Göttingen, Germany; 8 Bioanalytical Mass Spectrometry Group, Max Planck Institute for Multidisciplinary Sciences, 37075 Göttingen, Germany; 9 Institute for Animal Hygiene, Animal Welfare and Farm Animal Behaviour (ITTN), Stiftung Tierärztliche Hochschule Hannover, University of Veterinary Medicine, 30173 Hannover, Germany; 10 HCEMM-SU Cardiometabolic Immunology Research Group, Department of Pharmacology and Pharmacotherapy, Semmelweis University, H-1085 Budapest, Hungary; 11 Pharmahungary Group, H-1085 Budapest, Hungary; 12 Health and Medical University, D-14471 Potsdam, Germany

**Keywords:** Transcriptomics, Mechanisms of disease, Cardiac hypertrophy, Protein-protein interaction networks

Correction to: *Communications Biology* 10.1038/s42003-022-04402-9, published online 21 January 2023

The original version of the Article contained an error in Fig. 7b arising from the authors incorrectly cropping the image, leading to an inaccurate representation of the loading control.

This has now been corrected in the PDF and HTML versions of the Article.

Original figure:
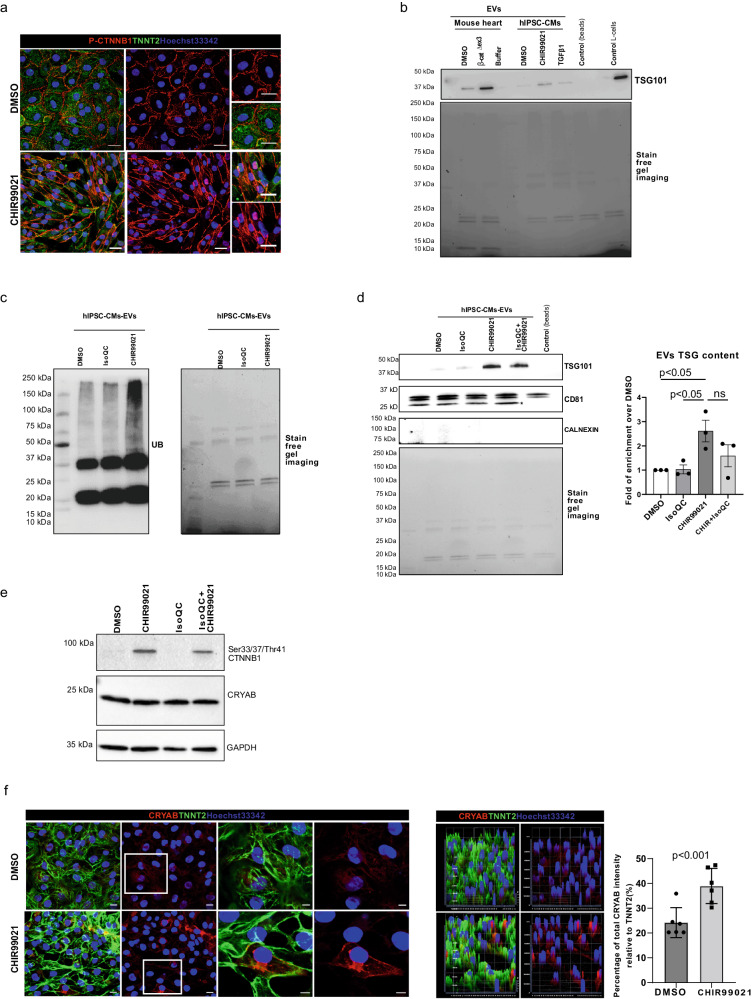


Corrected figure: