# Supplementary material for: Single-cell transcriptomics reveal extracellular vesicles secretion with a cardiomyocyte proteostasis signature during pathological remodeling
Source: Commun Biol. 2023 Jan 21;6:79. doi: 10.1038/s42003-022-04402-9 (PMC9867722; doi:10.1038/s42003-022-04402-9)
Supplement: Supplementary file 2 — Suplementary Figures [file 42003_2022_4402_MOESM2_ESM.pdf]

Supplementary Figures

Single-cell transcriptomics reveal extracellular vesicles secretion with a cardiomyocyte proteostasis signature during pathological remodeling

Eric Schoger[1,2,3]*, Federico Bleckwedel[1,2]*, Giulia Germena[4,2], Cheila Rocha[4], Petra Tucholla[1,2], Izzatullo Sobitov[1,2], Wiebke Möbius[5], Maren Sitte[6], Christof Lenz[7,8], Mostafa Samak[4,2], Rabea Hinkel[4,2,9], Zoltán V. Varga[10,11], Zoltán Giricz[10,11], Gabriela Salinas[6], Julia C. Gross[12] and Laura C. Zelarayán[1,2,3]
*The authors contributed equally

[1] Institute of Pharmacology and Toxicology, University Medical Center Göttingen (UMG), 37075 Göttingen, Germany
[2] German Center for Cardiovascular Research (DZHK) partner site Göttingen, 37075 Göttingen, Germany
[3] Cluster of Excellence "Multiscale Bioimaging: from Molecular Machines to Networks of Excitable Cells" (MBExC), University of Göttingen, 37075 Göttingen, Germany
[4] Laboratory Animal Science Unit, Leibnitz-Institut für Primatenforschung, Deutsches Primatenzentrum GmbH, 37075 Göttingen, Germany
[5] Max-Planck-Institute for Multidisciplinary Sciences, 37075 Göttingen, Germany
[6] NGS Integrative Genomics Core Unit (NIG), University Medical Center Göttingen (UMG), 37075 Göttingen, Germany
[7] Department of Clinical Chemistry, University Medical Center Göttingen (UMG), 37075 Göttingen, Germany
[8] Bioanalytical Mass Spectrometry Group, Max Planck Institute for Multidisciplinary Sciences, 37075 Göttingen, Germany
[9] Institute for Animal Hygiene, Animal Welfare and Farm Animal Behaviour (ITTN), Stiftung Tierärztliche Hochschule Hannover, University of Veterinary Medicine, 30173 Hannover, Germany.
[10] HCEMM-SU Cardiometabolic Immunology Research Group, Department of Pharmacology and Pharmacotherapy, Semmelweis University, H-1085 Budapest, Hungary
[11] Pharmahungary Group, H-1085 Budapest, Hungary
[12] Health and Medical University, D-14471 Potsdam, Germany

Corresponding Author: Laura C. Zelarayán, laura.zelarayan@med.uni-goettingen.de

Supplementary Figure 1

a

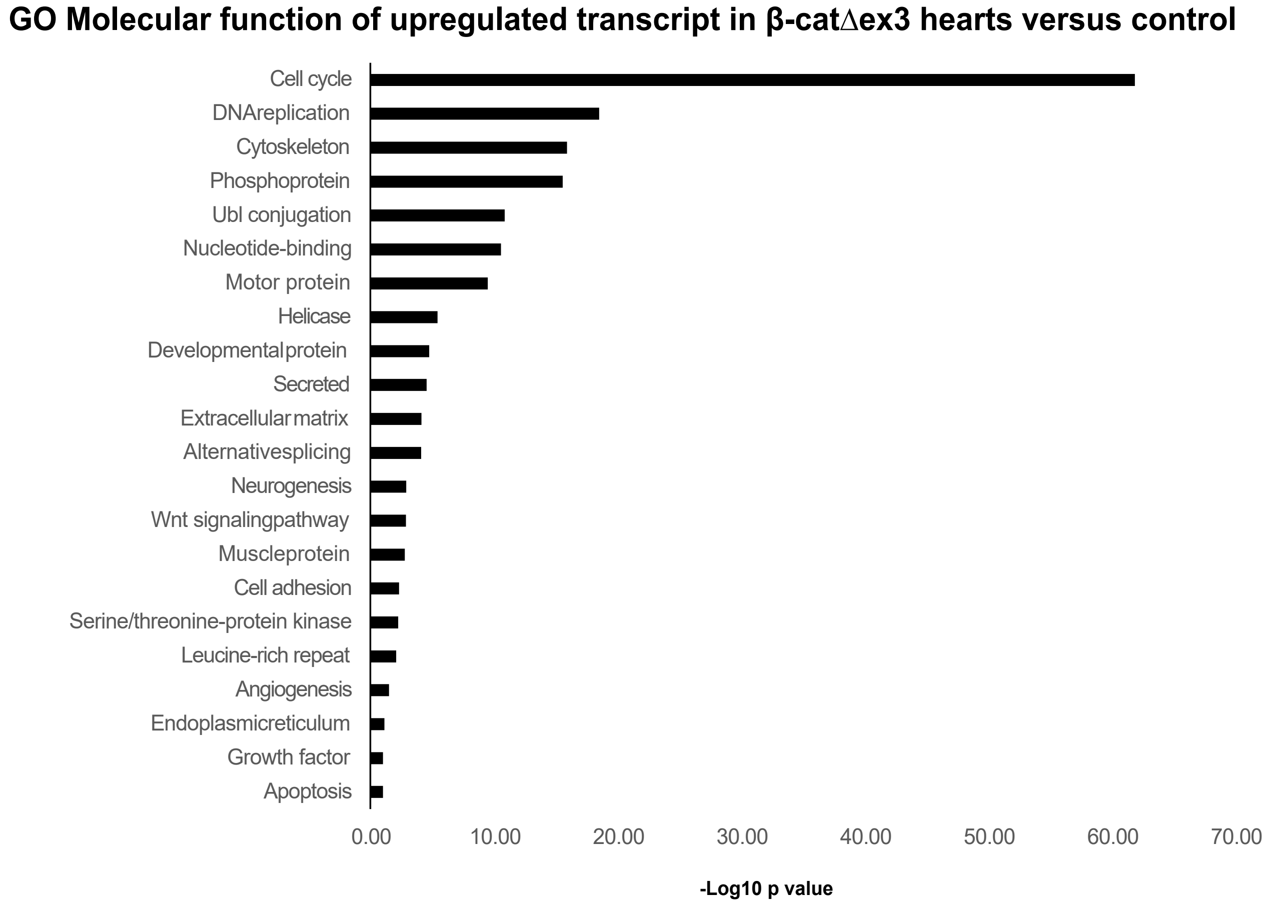

b

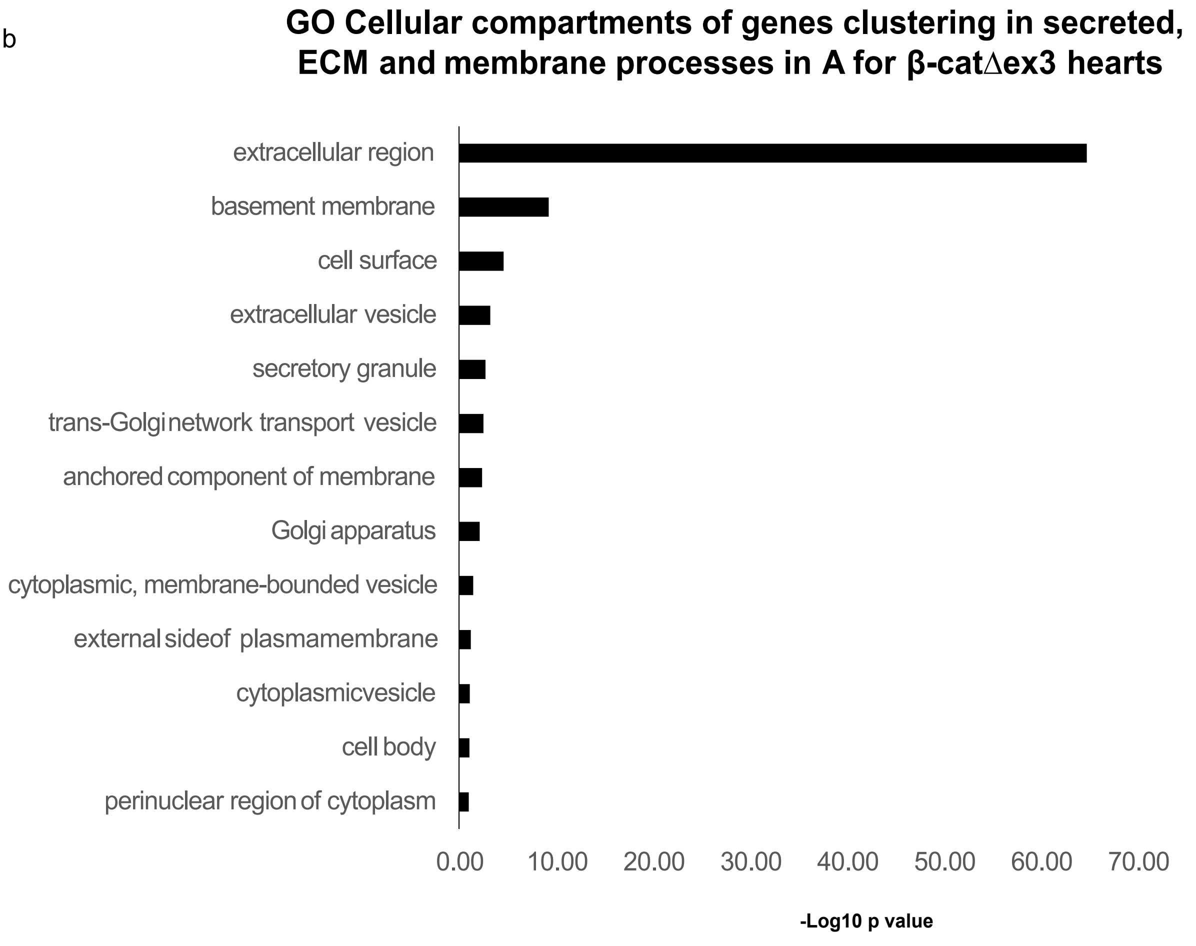

a. Selected top categories from gene ontology (GO) cellular compartments processes enrichment of upregulated DEGs in β-catΔex3 versus control hearts from bulk RNAseq data. **b**. Selected top categories from GO molecular function of genes clustering to membrane based processes and secreted proteins in **a**. –log10 p value (p value <0.05), term fusion was applied.

Supplementary Figure 2

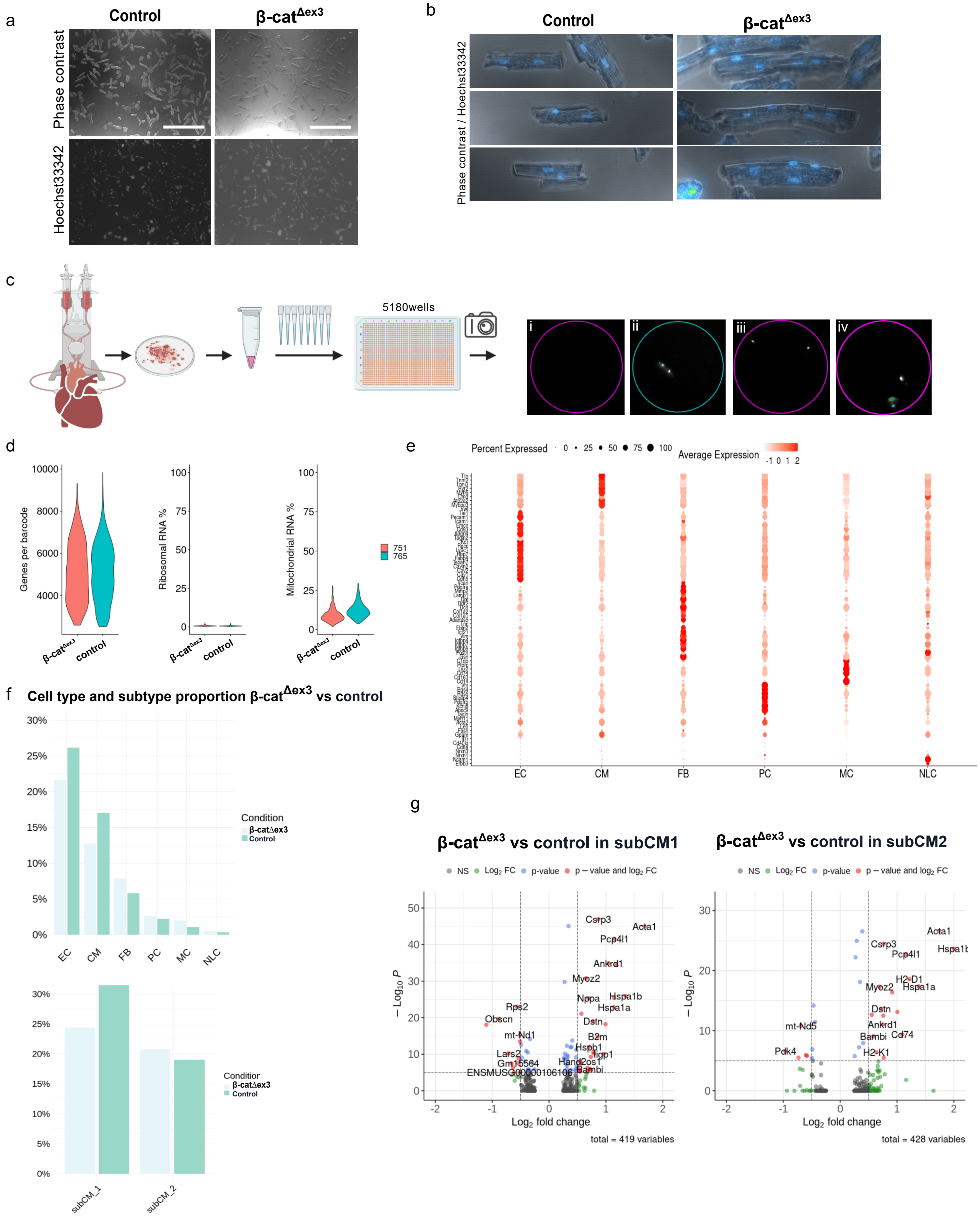

a. Isolated cardiac cells from Langendorf perfused hearts including rod-shaped cardiomyocytes and (**b**) hypernucleated, hypertrophic cardiomyocytes from β-cat$^{\Delta ex3}$ hearts compared to control. Scale bar = 20 µm. **c.** Schematic overview of cardiac cell isolation and distribution into nanowells for single cell transcriptome analyses using the iCell8 system. Cells are dissociated and dispensed into a 5180 well plate for library preparation and sequencing. The wells contained no cells (i), single multinucleated cells (ii), two cells (iii) or death cells (iv). Only cells as represented in **ii** were considered for sequencing. **d.** Quality control assessment of single cell transcripts analysed per heart indicating a mean of 4,5K and 4,4K genes per barcode (cell) in β-cat$^{\Delta ex3}$ and control samples respectively. In addition, low presence of ribosomal and mitochondrial contamination was confirmed. **e.** Dot plots showing different marker gene expression used to cluster cell-types according to known and reported cell markers. **f.** Distribution of cells in the different conditions (control or β-cat$^{\Delta ex3}$ hearts) as well as the distribution of the CM1 and CM2 subclusters. **g.** Volcano plot comparing DEGs in CM1 and CM2 β-cat$^{\Delta ex3}$ versus control hearts.

Supplementary Figure 3

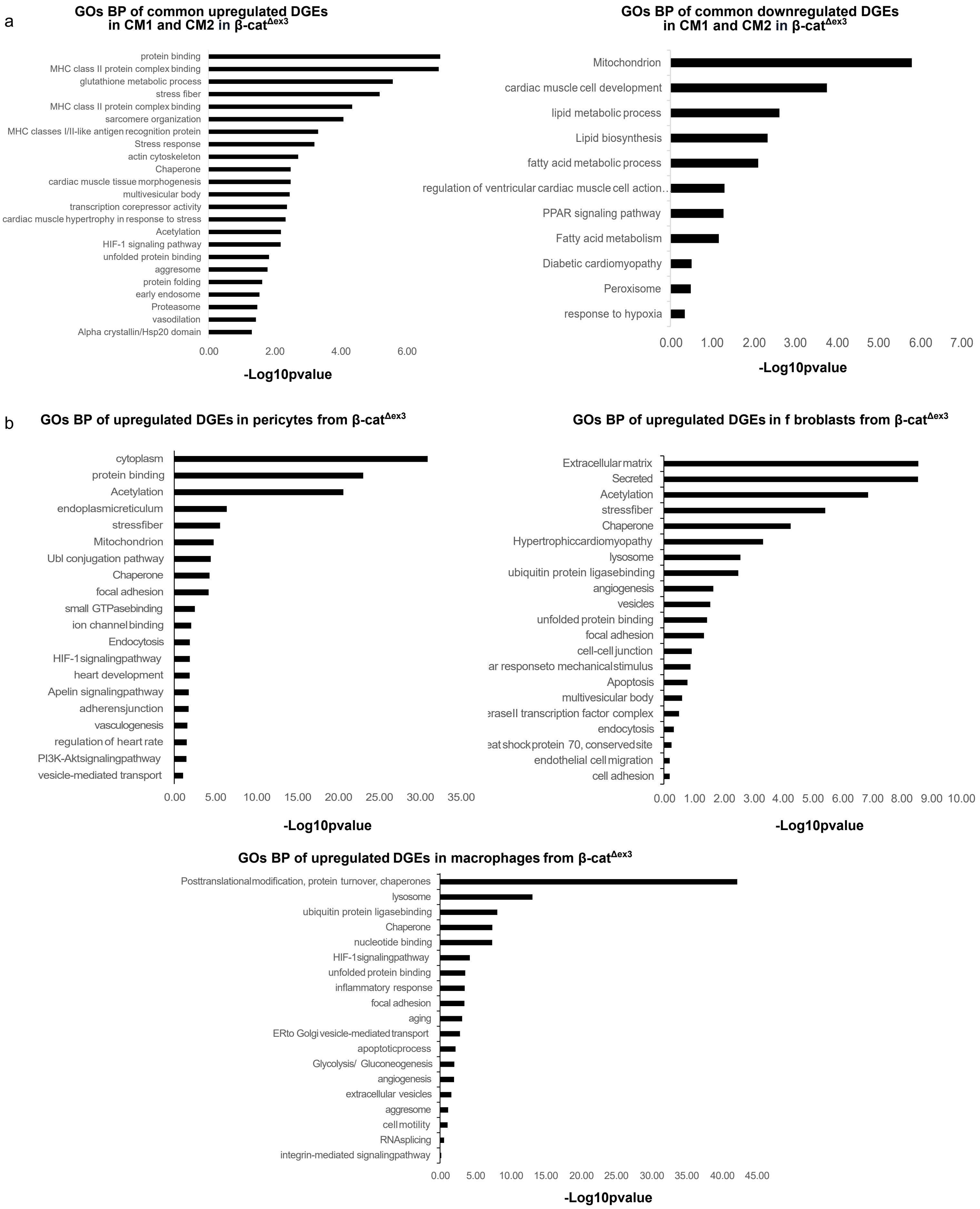

Selected top categories from gene ontology (GO) biological processes (BP) enrichment of common upregulated (left) and downregulated (right) DEGs in CM1 and CM2 (**a**) and in macrophage, fibroblast and pericyte clusters (**b**) of β-cat$^{\Delta ex3}$ versus control hearts. GO enrichment represents –log10 p-value (p-value <0.05) and term fusion was applied.

Supplementary Figure 4

a

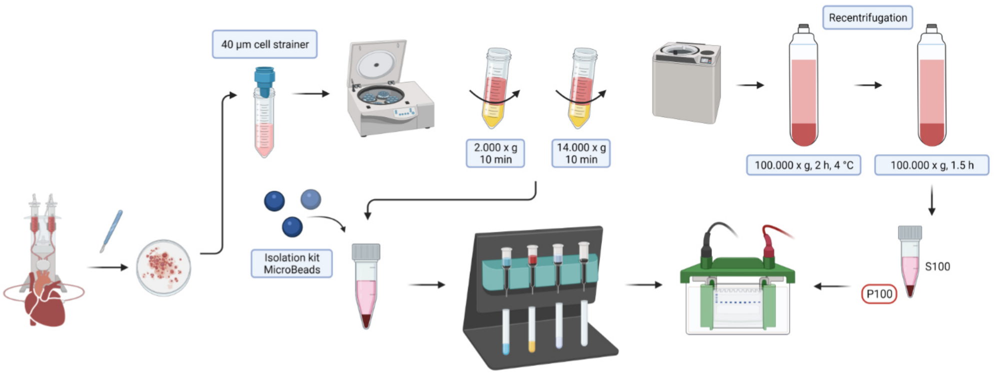

b

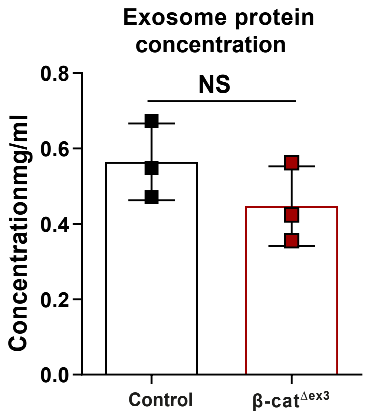

c

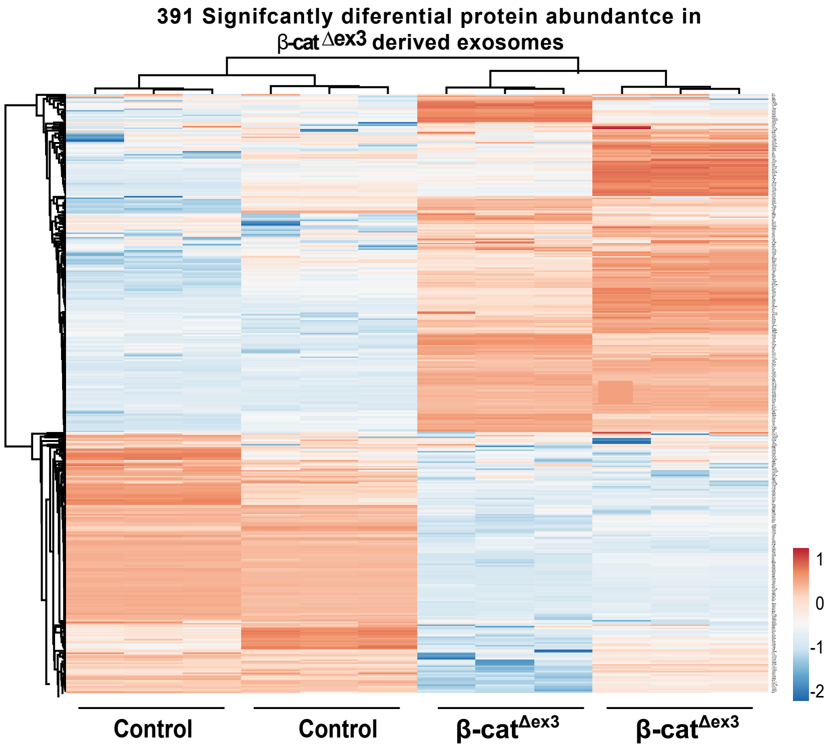

d

Exosomes proteins			
GeneID	Protein ID	Control	ß-cat $^{\Delta ex3}$
HSPA9	P38647	√	√
LDHA	P06151	√	√
PGK1	P00558	√	√
YWHAZ	P63101	√	√
HSP90AB1	P11499	√	√
YWHAE	P62259	√	√
CD47	Q61735	√	√
ENO1	P17182	√	√
CAV1	P49817	√	√
PKM	P52480	√	√
ALDOA	P05064	√	√
EEF1A2	P62631	√	√
HSPA8	P63017	√	√
CD81	P35762	√	√
Early endosomes			
GeneID	Protein ID	Control	ß-cat $^{\Delta ex3}$
VSP35	I1E4X5	√	√
CAV1	P49817	√	√
CAVIN2	Q63918	√	√
CAVIN1	O54724	√	√
CAVIN3	Q91VJ2	√	√
IGF2R	Q07113	√	√
RAB1A	P62821	√	√
LAMP2	P17047	√	√
CAV3	P51637	√	√

a. Overview of exosome isolation procedure from adult mouse hearts by UC and magnetic bead-based purification (Created with BioRender.com). **b.** Total protein concentration was comparable in exosome preparations regardless of experimental group (β-cat$^{\Delta ex3}$ and control). **c.** Heat-map of significantly enriched proteins (391) in β-cat$^{\Delta ex3}$ cardiac derived exosomes compared to controls. **d.** Classical exosomal and early endosomal proteins were identified enriched proteins.

Supplementary Figure 5

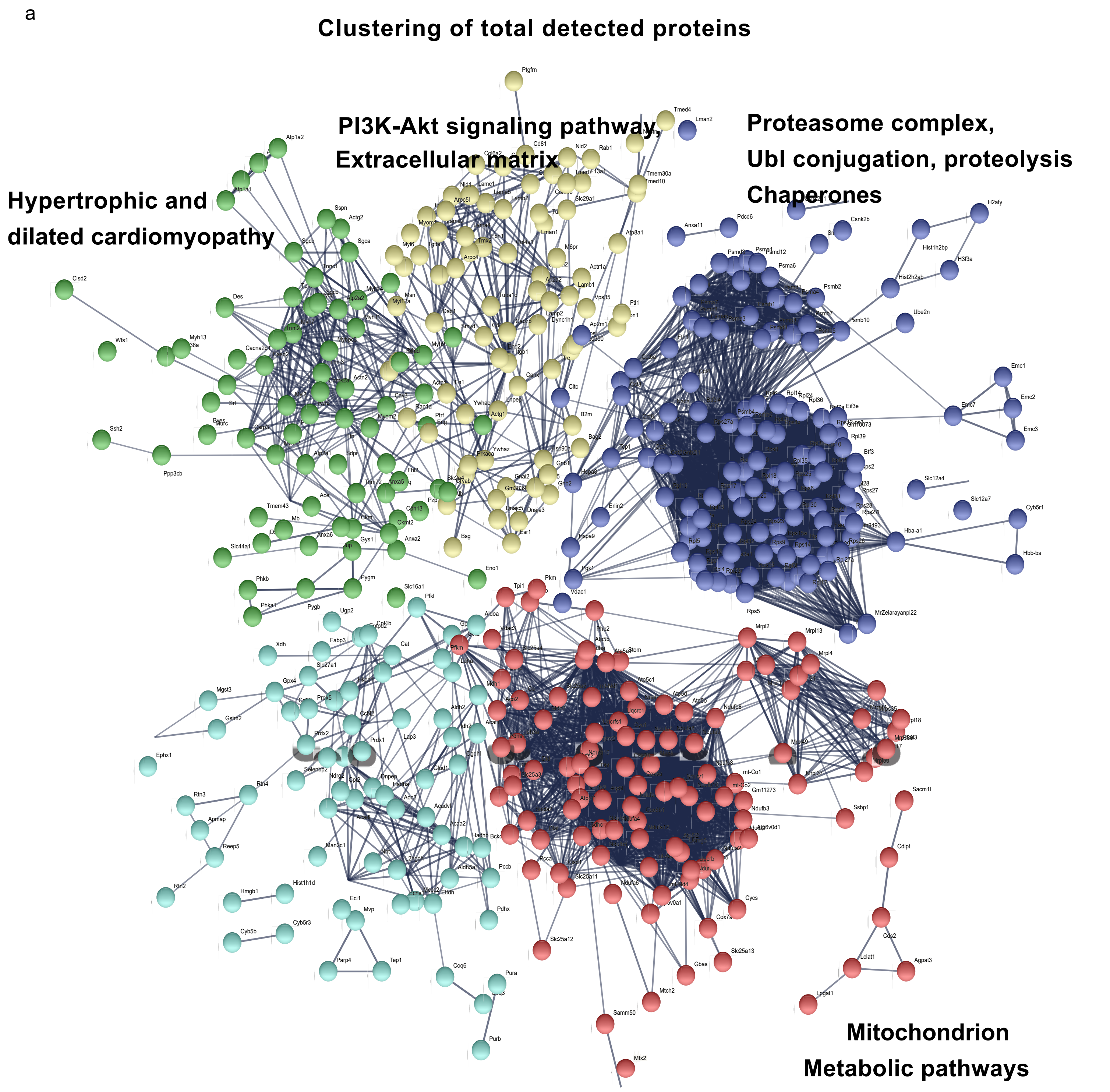

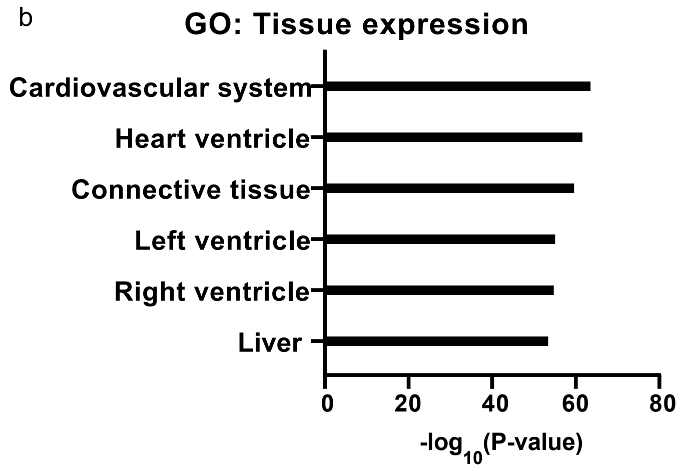

a. STRING analyses derived networks of protein-protein interactions of the total mass spectrometry data (573 proteins) revealed subclusters of protein classes with distinct cellular functions characteristic for EVs. **b.** The identified proteins categorized to the cardiovascular system in a tissue enrichment analysis. GO enrichment represents –log10 was considered for sequencing and term fusion was applied.

Supplementary Figure 6

a

Clustering of proteins enriched in β-cat$^{\Delta ex3}$isolated cardiac exosomes

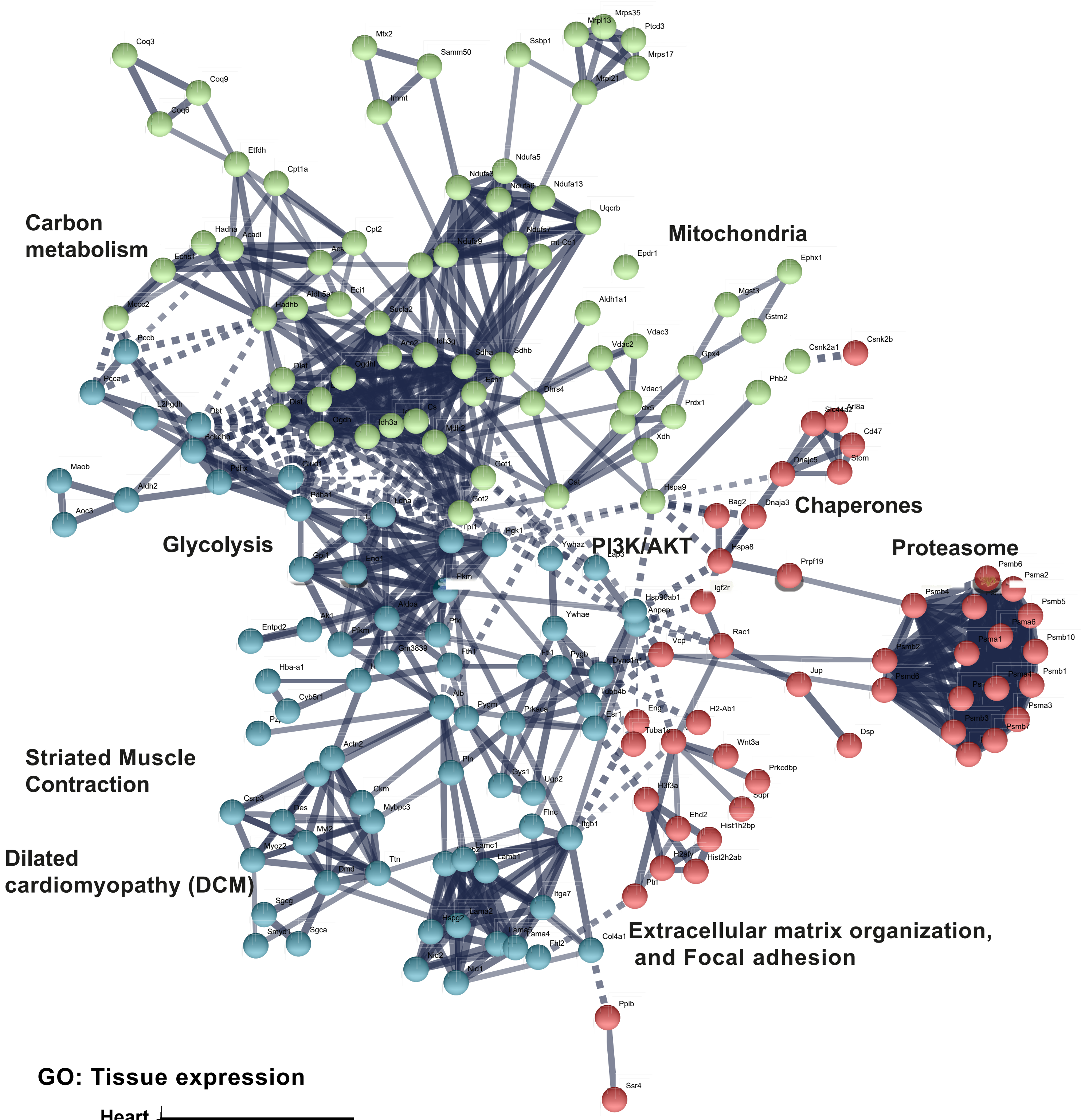

b

GO: Tissue expression

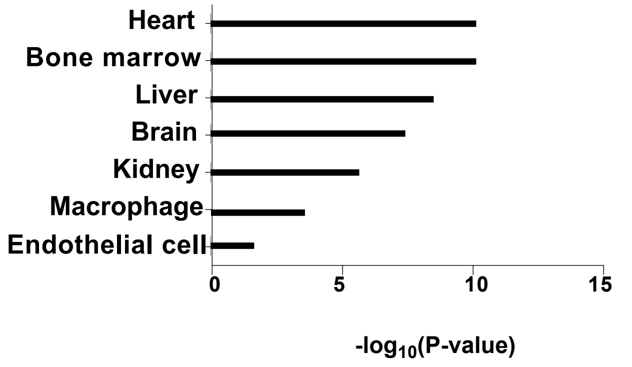

a. STRING analyses derived networks of protein-protein interactions of the enriched proteins (391) in β-cat$^{\Delta ex3}$ cardiac derived extracellular vesicles (EVs) compared to controls revealed sub-clusters of protein classes with distinct cellular functions characteristic for EVs. The identified proteins categorized to cardiovascular system in a tissue enrichment analysis. GO enrichment represents –log10 p-value (p-value <0.05) and term fusion was applied.

Supplementary Figure 7

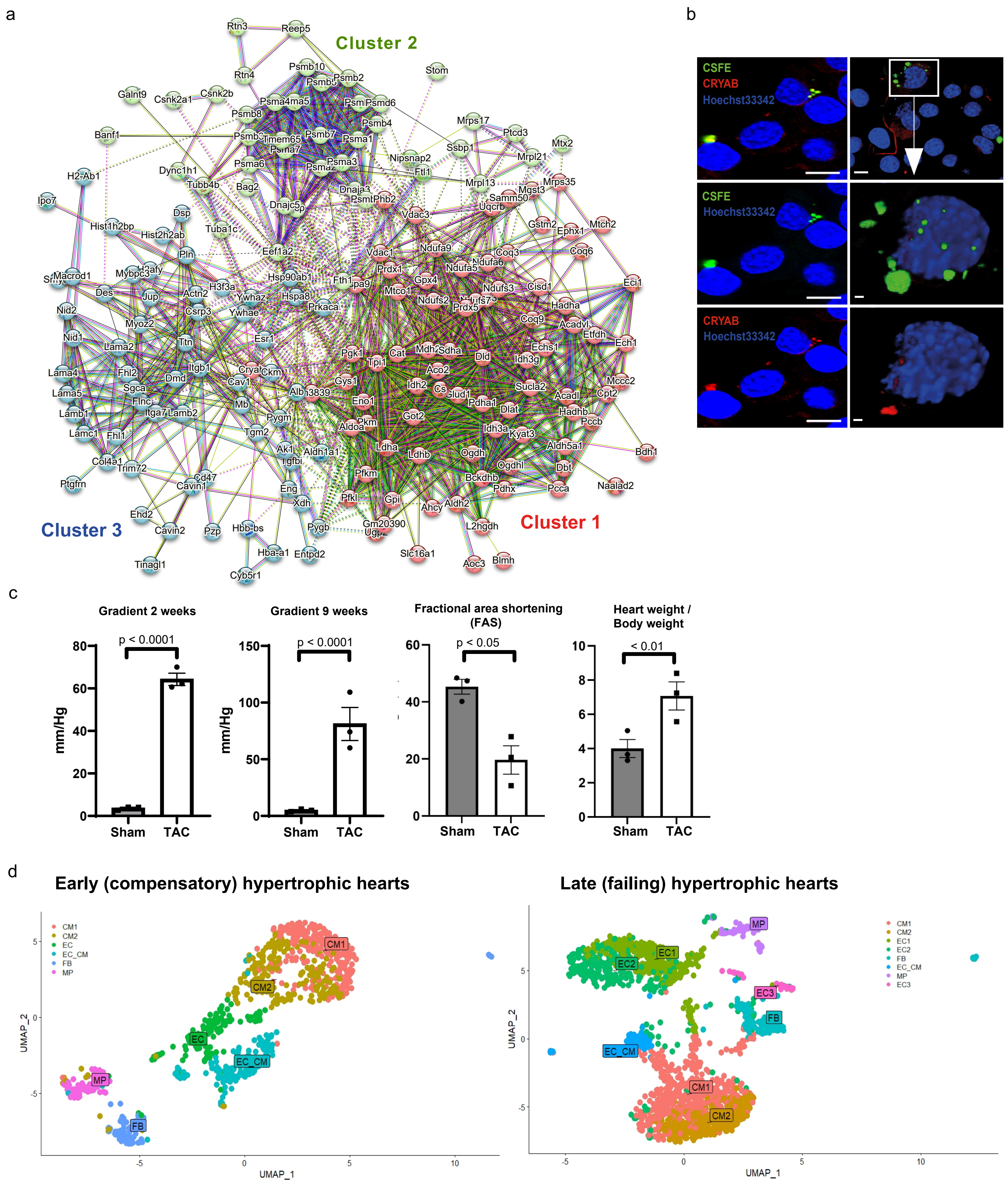

a. Cluster analysis using STRING functional annotation protein interaction database on the predicted enriched 391 proteins iin exosomes from β-cat$^{\Delta ex3}$ tissue using the K-means algorithm. They were summarized into three ontological clusters (cluster 1, 2 and 3). **b.** Representative confocal images of immunofluorescence staining of N2A neuroblastoma cells that were exposed to carboxyfluorescein succinimidyl ester (CSFE)-*ex vivo* labelled extracellular vesicles (EVs) from β-cat$^{\Delta ex3}$ hearts and stained with CRYAB (n=3, technical replicates). Hoechst33342 was used for nucleus visualization. By Z-scanning we observed that CRYAB co-localized with CFSE-labelled exosomes attached to the cell surface. Scale bar = 20 µm. **c**. Doppler-echo gradient confirming successful transverse aortic constriction with induced afterload increase upon intervention compared to sham in early- and late-stage disease condition. d. Fractional area shortening (FAS) was reduced upon TAC and heart weight normalized to body weight was elevated. Data are shown as mean ± SEM; t-*test*. **d**. UMAP plot after scRAN-seq and data integration of cardiac cells isolated from healthy (sham) and disease (TAC) hearts of CH (left) and FH (right) depicting the different cell clusters.

Supplementary Figure 8

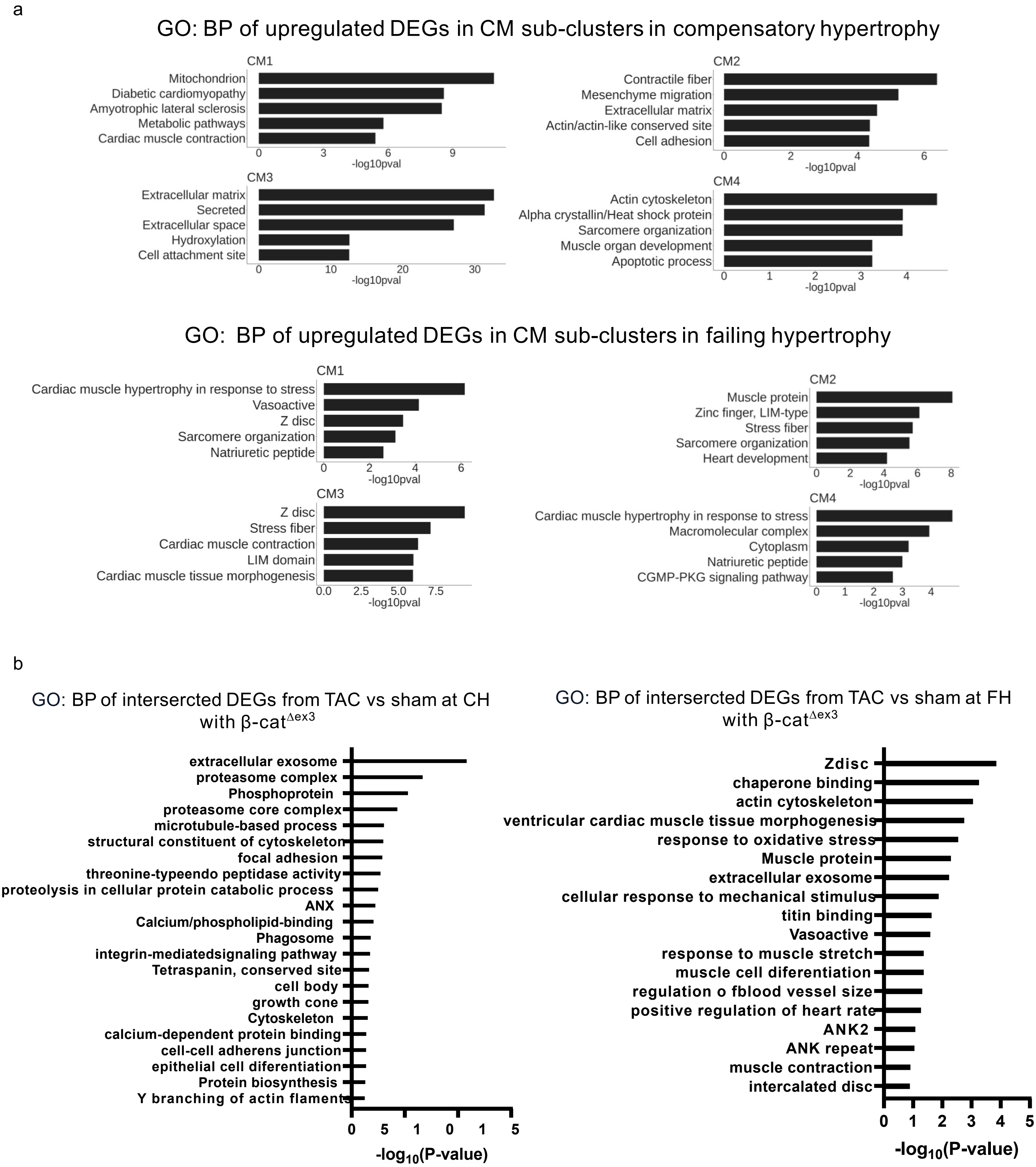

a. Selected top categories from gene ontology (GO) biological processes (BP) enrichment of upregulated DEGs in the different cardiomyocyte subclusters (CM1-CM4) from healthy (sham) and disease (TAC) hearts of compensatory and failing hypertrophy depicting the main transcriptional changes. **b**. Selected top categories from GO BP enrichment of overlapping upregulated DEGs in cardiomyocytes from TAC (versus sham) and β-cat$^{\Delta ex3}$ (versus control) of CH (left) and FH (right) depicting the main overlapping transcriptional changes at early and late disease states. GO enrichment represents –log10 p-value (p value <0.05) and term fusion was applied.

Supplementary Figure 9

a

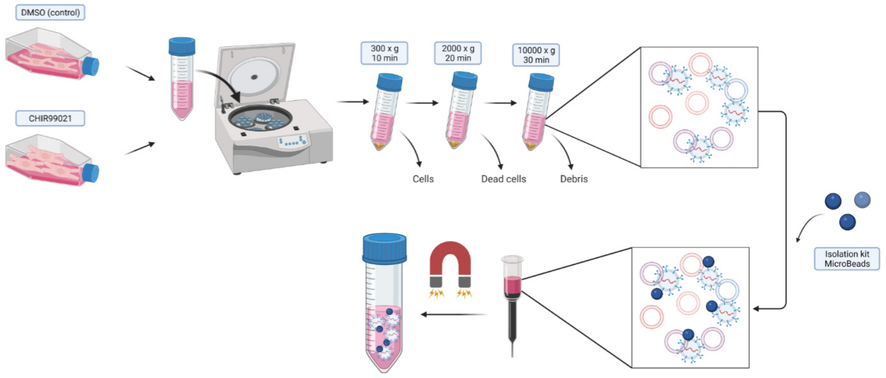

b

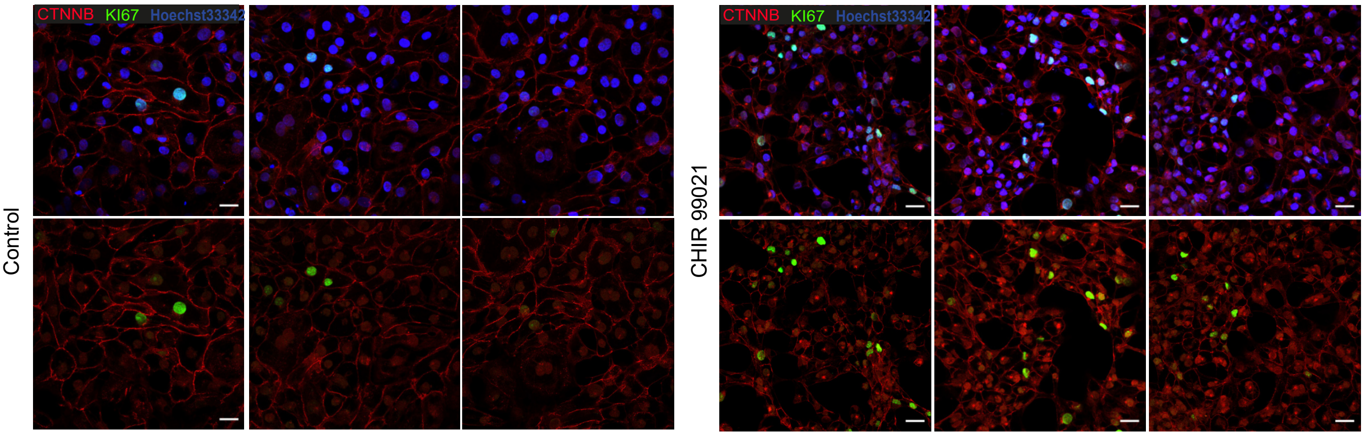

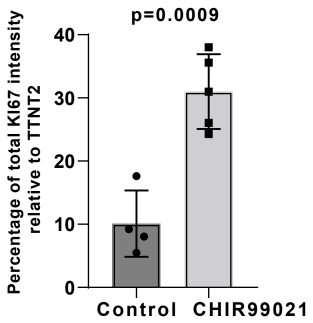

c

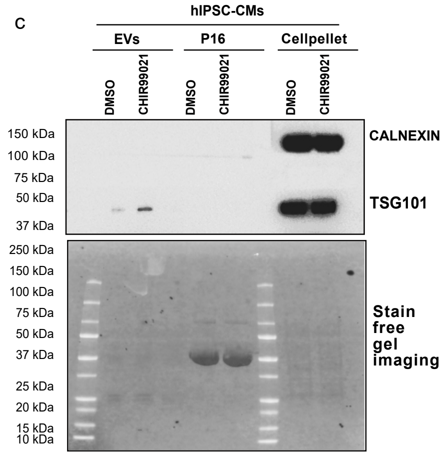

d

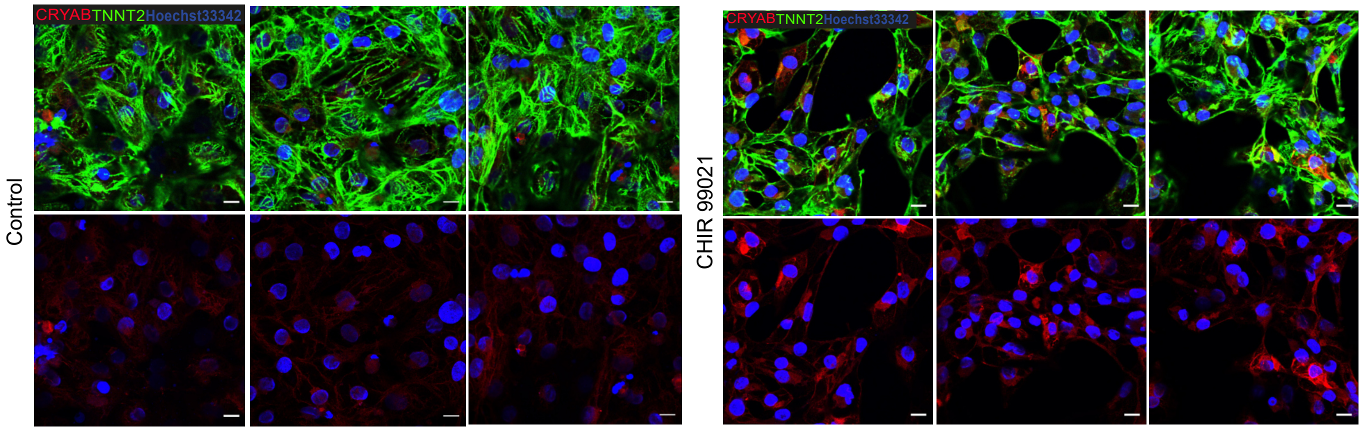

a. Schematic overview of extracellular vesicles (EVs) isolated from induced hiPSC-derived cardiomyocytes upon treatment with CHIR99021 (Wnt activated) or DMSO (control) using a magnetic bead-based protocol (n=9, technical replicates, biological duplicates) (Created with BioRender.com). **b.** Representative confocal images of immunofluorescence analysis showing increased cell cycle activity as assessed by KI67 staining and corresponding semiquantification in CHIR99021 treated hiPSC-derived cardiomyocytes versus control DMSO. (n=3, technical replicates). Scale bar = 20 µm. **c.** Western blot showing increased exosomal marker TSG101 as well as the absence of endoplasmic reticulum marker Calnexin in EVs from CHIR99021 treated hiPSC-derived cardiomyocytes. Calnexin was clearly detected in the cell pellet. Stain free gel showed equally amount of loaded proteins. **d.** Representative confocal images of immunofluorescence analysis showing CRYAB accumulation in CHIR99021 treated hiPSC-derived cardiomyocytes versus control DMSO. (n=3, technical replicates). Scale bar = 20 µm. Data are shown as mean ± SEM; unpaired student's t-*test*.

Supplementary Figure 10

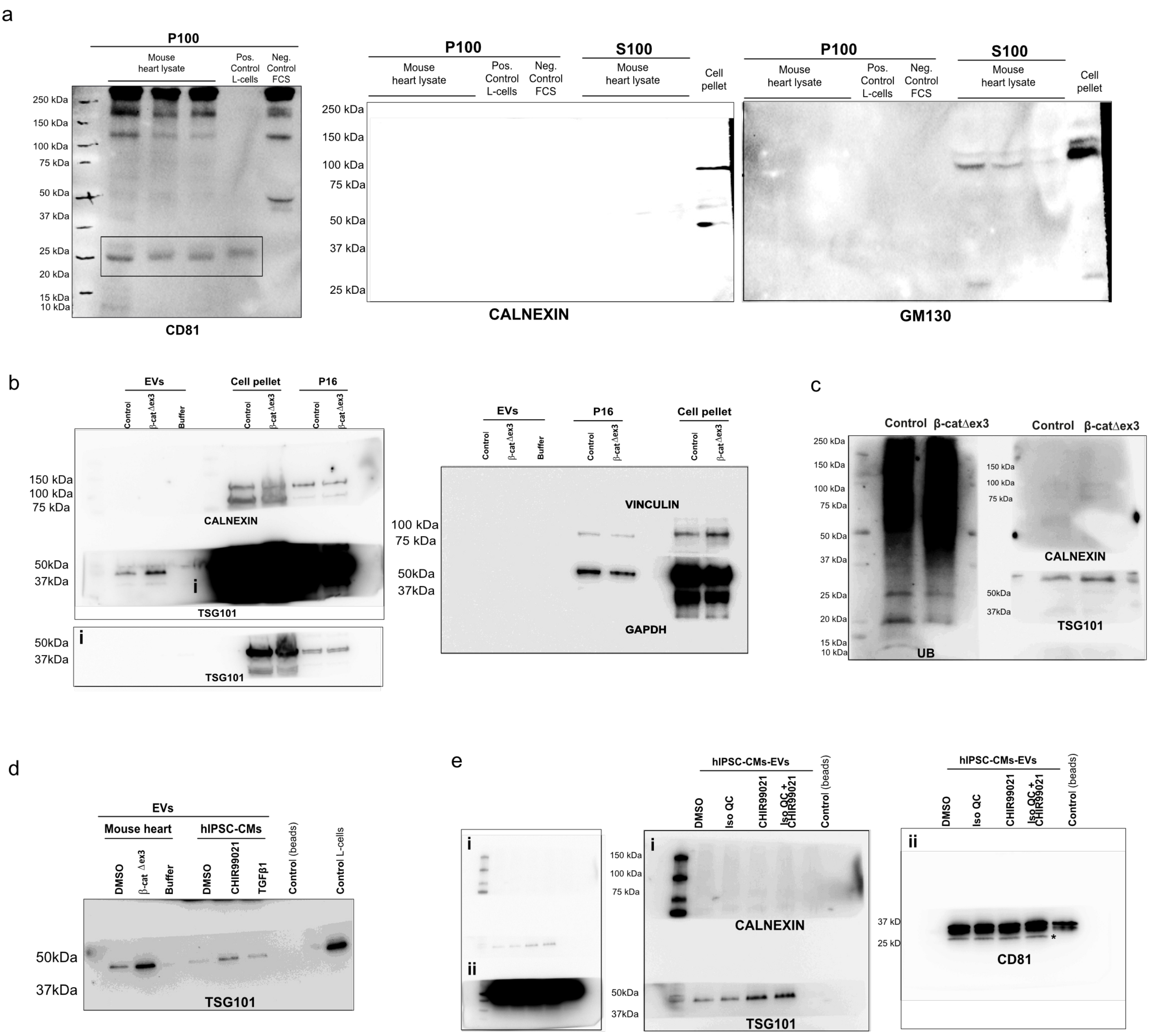

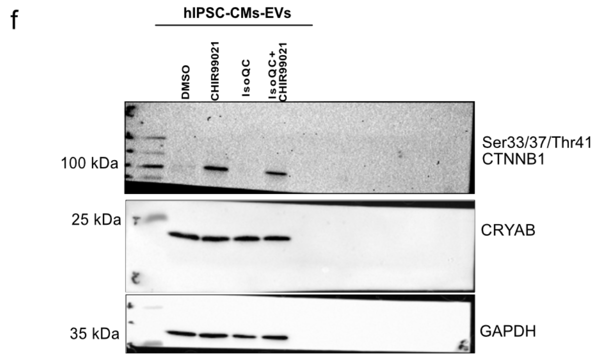

Whole blots used for the main figures in this study. **a**. Blots for Fig 2C. **b**. Blots for Fig 2D. (**i**, depicted below showing a reduced exposure time for TSG101). **c**. Blots for Fig 4A. **d**. Blots for Fig 7B. **e**. Blots for Fig 7D (**i**, shows an increased exposure time for Calnexin and TSG101 and **ii**, shows an reduced exposure time for CD81). **f**. Blots for Fig 7E.
