# Supplementary material for: Single-cell transcriptomics reveal extracellular vesicles secretion with a cardiomyocyte proteostasis signature during pathological remodeling
Source: Commun Biol. 2023 Jan 21;6:79. doi: 10.1038/s42003-022-04402-9 (PMC9867722; doi:10.1038/s42003-022-04402-9)
Supplement: Supplementary file 3 — Description of Additional Supplementary Files [file 42003_2022_4402_MOESM3_ESM.pdf]

Description of Additional Supplementary Files

File name: Supplementary Data 1
Description: ProteinPilot™ Software Report.

File name: Supplementary Data 2
Description: ExoCarta TOP proteins.

File name: Supplementary Data 3
Description: Antibody list.
