# Supplementary material for: Single-cell transcriptomics reveal extracellular vesicles secretion with a cardiomyocyte proteostasis signature during pathological remodeling
Source: Commun Biol. 2023 Jan 21;6:79. doi: 10.1038/s42003-022-04402-9 (PMC9867722; doi:10.1038/s42003-022-04402-9)
Supplement: Supplementary file 4 — Supplementary Data 1-3 [file 42003_2022_4402_MOESM4_ESM.pdf]

Supplementary Information

Single-cell transcriptomics reveal extracellular vesicles secretion with a cardiomyocyte proteostasis signature during pathological remodeling

Eric Schoger[1,2,3]*, Federico Bleckwedel[1,2]*, Giulia Germena[4,2], Cheila Rocha[4], Petra Tucholla[1,2], Izzatullo Sobitov[1,2], Wiebke Möbius[5], Maren Sitte[6], Christof Lenz[7], Mostafa Samak[4,2], Rabea Hinkel[4,2,8], Zoltán V. Varga[9,10], Zoltán Giricz[9,10], Gabriela Salinas[6], Julia C. Gross[11] and Laura C. Zelarayán[1,2,3]
*The authors contributed equally

[1] Institute of Pharmacology and Toxicology, University Medical Center Göttingen (UMG), 37075 Göttingen, Germany
[2] German Center for Cardiovascular Research (DZHK) partner site Göttingen, 37075 Göttingen, Germany
[3] Cluster of Excellence "Multiscale Bioimaging: from Molecular Machines to Networks of Excitable Cells" (MBExC), University of Göttingen, 37075 Göttingen, Germany
[4] Laboratory Animal Science Unit, Leibnitz-Institut für Primatenforschung, Deutsches Primatenzentrum GmbH, 37075 Göttingen, Germany
[5] Max-Planck-Institute for Multidisciplinary Sciences, 37075 Göttingen, Germany
[6] NGS Integrative Genomics Core Unit (NIG), University Medical Center Göttingen (UMG), 37075 Göttingen, Germany
[7] Department of Clinical Chemistry, University Medical Center Göttingen (UMG), 37075 Göttingen, Germany
[8] Bioanalytical Mass Spectrometry Group, Max Planck Institute for Multidisciplinary Sciences, 37075 Göttingen, Germany
[9] Institute for Animal Hygiene, Animal Welfare and Farm Animal Behaviour (ITTN), Stiftung Tierärztliche Hochschule Hannover, University of Veterinary Medicine, 30173 Hannover, Germany.
[10] HCEMM-SU Cardiometabolic Immunology Research Group, Department of Pharmacology and Pharmacotherapy, Semmelweis University, H-1085 Budapest, Hungary
[11] Pharmahungary Group, H-1085 Budapest, Hungary
[12] Health and Medical University, D-14471 Potsdam, Germany

Corresponding Author: Laura C. Zelarayán, laura.zelarayan@med.uni-goettingen.de

Supplementary Data 1

ProteinPilot™ Software Report
Template Version 1.00 light report
Summary of Identification Yields

	Data Level	FDR Type	FDR	ID Yield
Identification Yield at FDR Threshold	Protein	Local	1%	572
			5%	611
			10%	634
		Global	1%	656
			5%	737
			10%	810
	Distinct peptide	Local	1%	5821
			5%	6806
			10%	7224
		Global	1%	7330
			5%	8559
			10%	9437
	Spectral	Local	1%	68185
			5%	77518
			10%	81784
		Global	1%	83818
			5%	96643
			10%	98785
Corresponding Confidence in ProteinPilot™ Software	Protein	Local	1%	99.0%
			5%	95.9%
			10%	92.4%
		Global	1%	84.9%
			5%	46.3%
			10%	22.4%
	Distinct peptide	Local	1%	99.5%
			5%	97.1%
			10%	92.9%
		Global	1%	91.1%
			5%	49.9%
			10%	31.2%
	Spectral	Local	1%	97.2%
			5%	81.5%
			10%	62.0%
		Global	1%	49.8%
			5%	8.2%
			10%	0.1%

Total identified proteins: 573 Quants @ 1% FDR

Origin	Accession	ID	Gene names	Primary Gene
sp	A2ASS6	TITIN_MOUSE	Ttn	Ttn
sp	Q02566	MYH6_MOUSE	Myh6 Myhca	Myh6
sp	Q60675	LAMA2_MOUSE	Lama2	Lama2
sp	O55143-2	AT2A2_MOUSE	Atp2a2	Atp2a2
tr	F6U7V1	F6U7V1_MOUSE	Ryr2	Ryr2
sp	Q61554	FBN1_MOUSE	Fbn1 Fbn-1	Fbn1
tr	J3QQ16	J3QQ16_MOUSE	Col6a3	Col6a3
sp	Q9JI91	ACTN2_MOUSE	Actn2	Actn2
tr	E9Q3X0	E9Q3X0_MOUSE	Mvp	Mvp
tr	E9PZ16	E9PZ16_MOUSE	Hspg2	Hspg2
tr	Q3UHL6	Q3UHL6_MOUSE	Fn1	Fn1
sp	P58771	TPM1_MOUSE	Tpm1 Tpm-1 Tpma	Tpm1
sp	Q8BMF4	ODP2_MOUSE	Dlat	Dlat
sp	Q61292	LAMB2_MOUSE	Lamb2 Lams	Lamb2
sp	P68134	ACTS_MOUSE	Acta1 Acta	Acta1
tr	F8VQJ3	F8VQJ3_MOUSE	Lamc1	Lamc1
sp	Q8BMS1	ECHA_MOUSE	Hadha	Hadha
sp	Q04857	CO6A1_MOUSE	Col6a1	Col6a1
sp	Q01853	TERA_MOUSE	Vcp	Vcp
tr	D3Z041	D3Z041_MOUSE	Acsl1	Acsl1
tr	E9QN70	E9QN70_MOUSE	Lamb1	Lamb1
sp	Q8VDN2	AT1A1_MOUSE	Atp1a1	Atp1a1
tr	Q5SXR6	Q5SXR6_MOUSE	Cltc	Cltc
sp	P10493	NID1_MOUSE	Nid1 Ent	Nid1
sp	Q9D8E6	RL4_MOUSE	Rpl4	Rpl4
sp	Q61838	PZP_MOUSE	Pzp A2m	Pzp
sp	Q924X2	CPT1B_MOUSE	Cpt1b	Cpt1b
tr	E9QPE7	E9QPE7_MOUSE	Myh11	Myh11
sp	P09528	FRIH_MOUSE	Fth1 Fth	Fth1
sp	Q99JY0	ECHB_MOUSE	Hadhb	Hadhb
sp	Q60932	VDAC1_MOUSE	Vdac1 Vdac5	Vdac1
sp	P62702	RS4X_MOUSE	Rps4x Rps4	Rps4x
sp	P14148	RL7_MOUSE	Rpl7	Rpl7
sp	Q7TQ48	SRCA_MOUSE	Srl Sar	Srl
sp	Q9WUB3	PYGM_MOUSE	Pygm	Pygm
sp	P62908	RS3_MOUSE	Rps3	Rps3
tr	Q9CPX4	Q9CPX4_MOUSE	. Ftl1-ps1 mCG_17	Ftl1
sp	Q6P8J7	KCRS_MOUSE	Ckmt2	Ckmt2
sp	P48962	ADT1_MOUSE	Slc25a4 Anc1 Ant1	Slc25a4
tr	Z4YJV4	Z4YJV4_MOUSE	Ogdh	Ogdh
sp	Q8C129	LCAP_MOUSE	Lnpep	Lnpep
sp	P12970	RL7A_MOUSE	Rpl7a Surf-3 Surf3	Rpl7a
tr	F8WIT2	F8WIT2_MOUSE	Anxa6	Anxa6
sp	Q02788	CO6A2_MOUSE	Col6a2	Col6a2
sp	Q9D2G2	ODO2_MOUSE	Dlst	Dlst
tr	Q3UIK0	Q3UIK0_MOUSE	Mybpc3	Mybpc3
tr	A0A0R4J083	0A0R4J083_MOUS	Acadl	Acadl

sp	Q8VDD5	MYH9_MOUSE	Myh9	Myh9
sp	P97351	RS3A_MOUSE	Rps3a Rps3a1	Rps3a
sp	Q8BKZ9	ODPX_MOUSE	Pdhx	Pdhx
sp	Q03265	ATPA_MOUSE	Atp5f1a Atp5a1	Atp5f1a
tr	Q6P3Z7	Q6P3Z7_MOUSE	Tnnt2	Tnnt2
sp	P26041	MOES_MOUSE	Msn	Msn
sp	Q921G7	ETFD_MOUSE	Etfdh	Etfdh
tr	E9Q6A6	E9Q6A6_MOUSE	Col6a6	Col6a6
sp	P09542	MYL3_MOUSE	Myl3 Mlc1v Mylc	Myl3
sp	Q91VD9	NDUS1_MOUSE	Ndufs1	Ndufs1
sp	Q91ZJ5	UGPA_MOUSE	Ugp2	Ugp2
sp	P47857-3	PFKAM_MOUSE	Pfkm Pfk-m Pfka	Pfkm
sp	P14206	RSSA_MOUSE	Rpsa Lamr1 P40-8	Rpsa
sp	P47911	RL6_MOUSE	Rpl6	Rpl6
tr	A0A075B6A0	0A075B6A0_MOU	Ighm	Ighm
sp	P53395	ODB2_MOUSE	Dbt	Dbt
tr	E9Q1J7	E9Q1J7_MOUSE	Pccb	Pccb
tr	A0A1D5RLW5	0A1D5RLW5_MOU	Rpl18a	Rpl18a
sp	P47962	RL5_MOUSE	Rpl5	Rpl5
sp	P14869	RLA0_MOUSE	Rplp0 Arbp	Rplp0
sp	P14131	RS16_MOUSE	Rps16	Rps16
sp	Q1XH17	TRI72_MOUSE	Trim72 Mg53	Trim72
sp	Q8BLF1	NCEH1_MOUSE	eh1 Aadacl1 Kiaa1	Nceh1
sp	P25444	RS2_MOUSE	Rps2 Llrep3 Rps4	Rps2
sp	P68040	RACK1_MOUSE	ck1 Gnb2-rs1 Gnb	Rack1
sp	Q60930	VDAC2_MOUSE	Vdac2 Vdac6	Vdac2
tr	E9PWZ3	E9PWZ3_MOUSE	Rpl3l	Rpl3l
sp	P62242	RS8_MOUSE	Rps8	Rps8
tr	E9PYK3	E9PYK3_MOUSE	Parp4	Parp4
tr	Q921R2	Q921R2_MOUSE	ps13 mCG_12336	Rps13
sp	Q8K2B3	SDHA_MOUSE	Sdha	Sdha
sp	P56480	ATPB_MOUSE	Atp5f1b Atp5b	Atp5f1b
tr	A0A0R3P9C8	0A0R3P9C8_MOU	Ndufa9	Ndufa9
sp	Q91ZA3	PCCA_MOUSE	Pcca	Pcca
sp	P62918	RL8_MOUSE	Rpl8	Rpl8
sp	P61358	RL27_MOUSE	Rpl27	Rpl27
tr	Q5XJF6	Q5XJF6_MOUSE	mCG_123122 mCG	Rpl10a
sp	Q61001	LAMA5_MOUSE	Lama5	Lama5
sp	P07310	KCRM_MOUSE	Ckm Ckmm	Ckm
sp	O55234	PSB5_MOUSE	Psmb5	Psmb5
sp	P52825	CPT2_MOUSE	Cpt2 Cpt-2	Cpt2
sp	P63017	HSP7C_MOUSE	Hspa8 Hsc70 Hsc7	Hspa8
sp	O09061	PSB1_MOUSE	Psmb1	Psmb1
sp	Q6ZWN5	RS9_MOUSE	Rps9	Rps9
sp	P62245	RS15A_MOUSE	Rps15a	Rps15a
sp	Q9D1G3	HHATL_MOUSE	hatl Gup1 Kiaa117	Hhatl
sp	P19253	RL13A_MOUSE	l13a P198 Tstap19	Rpl13a
sp	P54071	IDHP_MOUSE	Idh2	Idh2
tr	Q3TVK3	Q3TVK3_MOUSE	Dnpep	Dnpep
sp	P52480-2	KPYM_MOUSE	km Pk3 Pkm2 Pyk	Pkm

sp	P05064	ALDOA_MOUSE	Aldoa Aldo1	Aldoa
sp	P62754	RS6_MOUSE	Rps6	Rps6
sp	Q6URW6-3	MYH14_MOUSE	Myh14	Myh14
sp	P82350	SGCA_MOUSE	Sgca	Sgca
tr	A0A0G2JES3	0A0G2JES3_MOUS	Rpl9	Rpl9
sp	Q64514	TPP2_MOUSE	Tpp2	Tpp2
sp	Q9R1P0	PSA4_MOUSE	Psma4	Psma4
sp	P47738	ALDH2_MOUSE	Aldh2 Ahd-1 Ahd1	Aldh2
sp	Q9Z2U0	PSA7_MOUSE	Psma7	Psma7
sp	Q9CZU6	CISY_MOUSE	Cs	Cs
sp	Q9CZ13	QCR1_MOUSE	Uqcrc1	Uqcrc1
sp	Q99KI0	ACON_MOUSE	Aco2	Aco2
tr	A0A1Y7VKY1	0A1Y7VKY1_MOU	n11361 mCG_116	Gm11361
tr	F6SVV1	F6SVV1_MOUSE	Gm9493	Gm9493
sp	Q9QUM9	PSA6_MOUSE	Psma6	Psma6
sp	P14094	AT1B1_MOUSE	Atp1b1 Atp4b	Atp1b1
sp	Q9R1P4	PSA1_MOUSE	Psma1	Psma1
sp	P35564	CALX_MOUSE	Canx	Canx
tr	A0A1B0GT92	A1B0GT92_MOU	Gys1	Gys1
tr	Q564E2	Q564E2_MOUSE	ha Ldh1 mCG_199	Ldha
tr	J3QMG3	J3QMG3_MOUSE	Vdac3	Vdac3
sp	P62889	RL30_MOUSE	Rpl30	Rpl30
sp	P11499	HS90B_MOUSE	ab1 Hsp84 Hsp84-1	Hsp90ab1
sp	Q99LB2	DHRS4_MOUSE	Dhrs4 D14Ucla2	Dhrs4
sp	P47963	RL13_MOUSE	Rpl13	Rpl13
sp	Q9CZX8	RS19_MOUSE	Rps19	Rps19
sp	Q9R118	HTRA1_MOUSE	Htra1 Htra Prss11	Htra1
sp	Q08857	CD36_MOUSE	Cd36	Cd36
tr	A0A1B0GQU8	A1B0GQU8_MOU	Rpl18	Rpl18
tr	E9QAZ2	E9QAZ2_MOUSE		
sp	Q9D0M3	CY1_MOUSE	Cyc1	Cyc1
sp	O54724	CAVN1_MOUSE	Cavin1 Ptrf	Cavin1
sp	Q9DB77	QCR2_MOUSE	Uqcrc2	Uqcrc2
sp	Q9DCT2	NDUS3_MOUSE	Ndufs3	Ndufs3
sp	P27659	RL3_MOUSE	Rpl3	Rpl3
sp	P62983	RS27A_MOUSE	s27a Uba80 Ubce	Rps27a
sp	Q9QXX4	CMC2_MOUSE	Slc25a13 Aralar2	Slc25a13
sp	O55126	NIPS2_MOUSE	Nipsnap2 Gbas	Nipsnap2
sp	P23927	CRYAB_MOUSE	Cryab Crya2	Cryab
sp	Q9CPR4	RL17_MOUSE	Rpl17	Rpl17
sp	P62281	RS11_MOUSE	Rps11	Rps11
sp	P19783	COX41_MOUSE	Cox4i1 Cox4 Cox4a	Cox4i1
sp	Q9D379	HYEP_MOUSE	Ephx1	Ephx1
sp	P20029	GRP78_MOUSE	Hspa5 Grp78	Hspa5
sp	Q9D517	PLCC_MOUSE	Agpat3 Lpaat3	Agpat3
sp	P26443	DHE3_MOUSE	Glud1 Glud	Glud1
sp	Q8CHS7	DRS7C_MOUSE	Dhrs7c Sdr32c2	Dhrs7c
sp	Q61941	NNTM_MOUSE	Nnt	Nnt
sp	Q9CQQ7	AT5F1_MOUSE	Atp5f1	Atp5f1
sp	Q9EQH3	VPS35_MOUSE	Vps35 Mem3	Vps35

sp	Q9Z2U1	PSA5_MOUSE	Psma5	Psma5
sp	P14115	RL27A_MOUSE	Rpl27a	Rpl27a
tr	Q3TWV4	Q3TWV4_MOUSE	p2m1 mCG_12845	Ap2m1
sp	Q9DCJ5	NDUA8_MOUSE	Ndufa8	Ndufa8
sp	P61255	RL26_MOUSE	Rpl26	Rpl26
sp	P63276	RS17_MOUSE	Rps17	Rps17
tr	D3YXT0	D3YXT0_MOUSE	Ndufs2	Ndufs2
sp	P62264	RS14_MOUSE	Rps14	Rps14
tr	A8DUK4	A8DUK4_MOUSE	Glna1 Hbb-bt Hbbt	Hbb-bs
sp	Q62351	TFR1_MOUSE	Tfrc Trfr	Tfrc
sp	P62806	H4_MOUSE	4f; Hist1h4h; Hist1	Hist1h4a;
sp	P12382	PFKAL_MOUSE	Pfkl Pfk-l Pfkb	Pfkl
sp	O70435	PSA3_MOUSE	Psma3	Psma3
sp	Q9D710	TMX2_MOUSE	Tmx2 Txndc14	Tmx2
tr	D3YUM1	D3YUM1_MOUSE	Ndufv1	Ndufv1
sp	O35129	PHB2_MOUSE	hb2 Bap Bcap37 Re	Phb2
tr	Q91V55	Q91V55_MOUSE	Rps5 mCG_22552	Rps5
sp	P62631	EF1A2_MOUSE	Eef1a2 Eef1al Stn	Eef1a2
sp	P47754	CAZA2_MOUSE	Capza2 Cappa2	Capza2
sp	P49722	PSA2_MOUSE	Psma2 Lmpc3	Psma2
tr	Q6ZWZ6	Q6ZWZ6_MOUSE	ps12 mCG_132913	Rps12-ps3
sp	P48036	ANXA5_MOUSE	Anxa5 Anx5	Anxa5
sp	P24270	CATA_MOUSE	Cat Cas-1 Cas1	Cat
sp	Q9R1P3	PSB2_MOUSE	Psmb2	Psmb2
sp	P63260	ACTG_MOUSE	Actg1 Actg	Actg1
sp	P67984	RL22_MOUSE	Rpl22	Rpl22
sp	Q60994	ADIPO_MOUSE	poq Acdc Acrp30 A	Adipoq
tr	J3QP71	J3QP71_MOUSE	Bsg	Bsg
sp	Q8BP67	RL24_MOUSE	Rpl24	Rpl24
tr	A0A1L1SQA8	0A1L1SQA8_MOUS	Rps25	Rps25
sp	Q60692	PSB6_MOUSE	Psmb6 Lmp19	Psmb6
sp	P16125	LDHB_MOUSE	Ldhb Ldh-2 Ldh2	Ldhb
tr	Q9CQB4	Q9CQB4_MOUSE	Uqcrb mCG_67985	Uqcrb
tr	A0A140T8M7	0A140T8M7_MOUSE		
sp	Q8CGP2	H2B1P_MOUSE	Hist1h2bp	Hist1h2bp
sp	Q8R1S0	COQ6_MOUSE	Coq6	Coq6
sp	Q9DBH5	LMAN2_MOUSE	Lman2	Lman2
tr	F2Z456	F2Z456_MOUSE	Cyb5r3	Cyb5r3
sp	P62900	RL31_MOUSE	Rpl31	Rpl31
sp	P09055	ITB1_MOUSE	Itgb1	Itgb1
sp	P05202	AATM_MOUSE	Got2 Got-2	Got2
tr	B0V2N8	B0V2N8_MOUSE	Anxa2	Anxa2
sp	Q8BWF0	SSDH_MOUSE	Aldh5a1	Aldh5a1
tr	H3BL49	H3BL49_MOUSE	Cct8	Cct8
sp	Q9CR62	M2OM_MOUSE	Slc25a11	Slc25a11
sp	Q8CI94	PYGB_MOUSE	Pygb	Pygb
sp	Q9D1D4	TMEDA_MOUSE	Tmed10 Tmp21	Tmed10
tr	Q9CQM8	Q9CQM8_MOUSE	nCG_121646 mCG_	Rpl21
sp	Q91YQ5	RPN1_MOUSE	Rpn1	Rpn1
sp	Q6PIE5	AT1A2_MOUSE	Atp1a2	Atp1a2

tr	G5E902	G5E902_MOUSE	lc25a3 mCG_1034	Slc25a3
sp	P70195	PSB7_MOUSE	Psmb7 Mmc14	Psmb7
tr	Q91VB8	Q91VB8_MOUSE	obin alpha 1 haem	Hba-a1
sp	O70572	NSMA_MOUSE	Smpd2	Smpd2
sp	Q8BW75	AOFB_MOUSE	Maob	Maob
tr	E9PZF0	E9PZF0_MOUSE	Gm20390	Gm20390
sp	P62911	RL32_MOUSE	Rpl32	Rpl32
sp	P35979	RL12_MOUSE	Rpl12	Rpl12
sp	Q9R1P1	PSB3_MOUSE	Psmb3	Psmb3
sp	Q60737	CSK21_MOUSE	Csnk2a1 Ckiia	Csnk2a1
tr	Q6ZWZ4	Q6ZWZ4_MOUSE	Rpl36 mCG_20352	Rpl36
sp	Q99MQ4	ASPN_MOUSE	Aspn	Aspn
sp	P11404	FABPH_MOUSE	Fabp3 Fabph1	Fabp3
tr	A0A0A0MQG2	A0A0MQG2_MOU	Sptbn1	Sptbn1
tr	I7HLV2	I7HLV2_MOUSE	10 RP23-436K3.4-(	Rpl10
tr	G3X9L6	G3X9L6_MOUSE		
tr	S4R1W1	S4R1W1_MOUSE	Gm3839	Gm3839
sp	Q80XN0	BDH_MOUSE	Bdh1 Bdh	Bdh1
sp	P99026	PSB4_MOUSE	Psmb4 Lmp3	Psmb4
sp	Q9D0G0	RT30_MOUSE	Mrps30	Mrps30
sp	Q6ZWV7	RL35_MOUSE	Rpl35	Rpl35
sp	P80318	TCPG_MOUSE	Cct3 Cctg	Cct3
sp	Q60817	NACA_MOUSE	Naca	Naca
tr	A0A286YEB7	0A286YEB7_MOUS	Rps24	Rps24
sp	Q9CR57	RL14_MOUSE	Rpl14	Rpl14
sp	P07724	ALBU_MOUSE	Alb Alb-1 Alb1	Alb
tr	A0A0N4SV00	0A0N4SV00_MOUS	Cct7	Cct7
tr	A0A1W2P7Q9	A1W2P7Q9_MOU	Myl6	Myl6
sp	Q63918	CAVN2_MOUSE	Cavin2 Sdpr Sdr	Cavin2
tr	A0A0J9YUZ4	0A0J9YUZ4_MOUS	Hmgb1	Hmgb1
sp	Q91Z83	MYH7_MOUSE	Myh7	Myh7
sp	P14142	GTR4_MOUSE	Slc2a4 Glut-4 Glut4	Slc2a4
sp	Q3UN02	LCLT1_MOUSE	at1 Alcat1 Gm91 Ly	Lclat1
sp	P62855	RS26_MOUSE	Rps26	Rps26
tr	A0A0G2JDL9	0A0G2JDL9_MOUS	Rap1a mCG_10748	Rap1a
sp	O54734	OST48_MOUSE	Ddost	Ddost
tr	Q3TLP8	Q3TLP8_MOUSE	Rac1 mCG_23557	Rac1
sp	O55026	ENTP2_MOUSE	Entpd2 Cd39l1	Entpd2
tr	B1AR69	B1AR69_MOUSE	Myh13	Myh13
sp	Q9CPY7	AMPL_MOUSE	Lap3 Lapep	Lap3
sp	Q91YN9	BAG2_MOUSE	Bag2	Bag2
sp	P97429	ANXA4_MOUSE	Anxa4 Anx4	Anxa4
sp	P08752	GNAI2_MOUSE	Gnai2 Gnai-2	Gnai2
sp	P51637	CAV3_MOUSE	Cav3	Cav3
sp	P56391	CX6B1_MOUSE	Cox6b1 Cox6b	Cox6b1
sp	P67778	PHB_MOUSE	Phb	Phb
tr	Q3UW83	Q3UW83_MOUSE	Rps10	Rps10
sp	Q7TSH2	KPBB_MOUSE	Phkb	Phkb
tr	Q9D881	Q9D881_MOUSE	ox5b mCG_17741	Gm11273
sp	P62830	RL23_MOUSE	Rpl23	Rpl23

sp	P61164	ACTZ_MOUSE	Actr1a Ctrn1	Actr1a
sp	P47757	CAPZB_MOUSE	Capzb Cappb1	Capzb
sp	Q9DB20	ATPO_MOUSE	Atp5o D12Wsu28e	Atp5o
tr	E9Q7G1	E9Q7G1_MOUSE	Tmed7	Tmed7
sp	Q9R069	BCAM_MOUSE	Bcam Gplu Lu	Bcam
tr	G3X8R0	G3X8R0_MOUSE	?eep5 mCG_12149	Reep5
sp	P52503	NDUS6_MOUSE	Ndufs6 Ip13	Ndufs6
sp	P04247	MYG_MOUSE	Mb	Mb
sp	P51881	ADT2_MOUSE	Slc25a5 Ant2	Slc25a5
sp	Q8R127	SCPDL_MOUSE	Sccpdh	Sccpdh
sp	Q8C7X2	EMC1_MOUSE	Emc1 Kiaa0090	Emc1
sp	Q9D1N9	RM21_MOUSE	Mrpl21 D9Wsu149	Mrpl21
sp	O35459	ECH1_MOUSE	Ech1	Ech1
sp	P28063	PSB8_MOUSE	Psmb8 Lmp7 Mc13	Psmb8
sp	Q3TMP8	TM38A_MOUSE	Tmem38a	Tmem38a
sp	P41105	RL28_MOUSE	Rpl28	Rpl28
sp	P14685	PSMD3_MOUSE	smd3 P91a Tstap91	Psmd3
sp	E9Q557	DESP_MOUSE	Dsp	Dsp
sp	Q8BH64	EHD2_MOUSE	Ehd2	Ehd2
sp	Q9ERS2	NDUAD_MOUSE	Ndufa13 Grim19	Ndufa13
tr	S4R1E5	S4R1E5_MOUSE	Gpx4	Gpx4
tr	E9Q8P0	E9Q8P0_MOUSE	Tnnc1	Tnnc1
sp	P48787	TNNI3_MOUSE	Tnni3	Tnni3
sp	Q9CQB5	CISD2_MOUSE	2 Cdgsh2 Noxp70	Cisd2
sp	Q922B1	MACD1_MOUSE	Macrod1 Lrp16	Macrod1
sp	P82347	SGCD_MOUSE	Sgcd	Sgcd
sp	P63268	ACTH_MOUSE	Actg2 Acta3 Actsg	Actg2
sp	O88322	NID2_MOUSE	Nid2	Nid2
tr	F6QYE1	F6QYE1_MOUSE	Casq2	Casq2
sp	Q9CPU4	MGST3_MOUSE	Mgst3	Mgst3
sp	P31428	DPEP1_MOUSE	Dpep1 Mbd1 Rdp	Dpep1
sp	Q9DCS9	NDUBA_MOUSE	Ndufb10	Ndufb10
tr	Q3TZS3	Q3TZS3_MOUSE	Itga7	Itga7
tr	Q9D050	Q9D050_MOUSE	Mtch2	Mtch2
sp	P97384	ANX11_MOUSE	Anxa11 Anx11	Anxa11
sp	Q9CQA3	SDHB_MOUSE	Sdhb	Sdhb
sp	Q8BMS4	COQ3_MOUSE	Coq3	Coq3
tr	Q5SVW9	Q5SVW9_MOUSE	Tmed4	Tmed4
sp	Q9ES97	RTN3_MOUSE	Rtn3	Rtn3
sp	P62897	CYC_MOUSE	Cycs	Cycs
sp	P12787	COX5A_MOUSE	Cox5a	Cox5a
sp	O70622	RTN2_MOUSE	Rtn2 Nspl1	Rtn2
tr	D3YTQ9	D3YTQ9_MOUSE	Rps15	Rps15
tr	A0A0N4SW94	)A0N4SW94_MOU	Myadm	Myadm
tr	A0A0G2JG29	)A0G2JG29_MOUS	Rps27	Rps27
sp	Q9CQZ5	NDUA6_MOUSE	Ndufa6	Ndufa6
sp	Q99JB8	PACN3_MOUSE	Pacsin3	Pacsin3
sp	Q64522	H2A2B_MOUSE	Hist2h2ab	Hist2h2ab
sp	P06745	G6PI_MOUSE	Gpi Gpi1	Gpi
sp	P00405	COX2_MOUSE	:o2 COII COX2 mt-(	Mtco2

sp	A2AMM0	CAVN4_MOUSE	Cavin4 Murc	Cavin4
sp	Q9D1P0	RM13_MOUSE	Mrpl13	Mrpl13
sp	P70704	AT8A1_MOUSE	Atp8a1 Atpc1	Atp8a1
sp	Q9CR61	NDUB7_MOUSE	Ndufb7	Ndufb7
tr	E9Q1X8	E9Q1X8_MOUSE	Cacna2d1	Cacna2d1
tr	G5E839	G5E839_MOUSE	Cct4	Cct4
sp	Q9QYG0	NDRG2_MOUSE	drg2 Kiaa1248 Ndı	Ndrg2
tr	H3BJQ7	H3BJQ7_MOUSE	Prdx5	Prdx5
sp	P43277	H13_MOUSE	Hist1h1d H1f3	Hist1h1d
sp	Q99LC3	NDUAA_MOUSE	Ndufa10	Ndufa10
sp	Q64152	BTF3_MOUSE	Btf3	Btf3
sp	P59266	FITM2_MOUSE	Fitm2 Fit2	Fitm2
sp	P31001	DESM_MOUSE	Des	Des
sp	P08249	MDHM_MOUSE	Mdh2 Mor1	Mdh2
tr	E0CZ27	E0CZ27_MOUSE	H3f3a	H3f3a
sp	P54116	STOM_MOUSE	Stom Epb7.2 Epb72	Stom
tr	G3UXL2	G3UXL2_MOUSE	Prps1l3	Prps1l3
sp	P17751	TPIS_MOUSE	Tpi1 Tpi	Tpi1
sp	Q9WV91	FPRP_MOUSE	Ptgfrn Fprp	Ptgfrn
sp	Q60714	S27A1_MOUSE	Slc27a1 Fatp Fatp1	Slc27a1
sp	Q9CPP6	NDUA5_MOUSE	Ndufa5	Ndufa5
sp	Q8K3J1	NDUS8_MOUSE	Ndufs8	Ndufs8
sp	Q9EP89	LACTB_MOUSE	Lactb Lact1	Lactb
sp	P80317	TCPZ_MOUSE	ct6a Cct6 Cctz Cctz	Cct6a
sp	P09470	ACE_MOUSE	Ace Dcp1	Ace
sp	Q06185	ATP5I_MOUSE	p5i Atp5k Lfm-1 Lfı	Atp5i
sp	P03911	NU4M_MOUSE	Mtnd4 mt-Nd4 Nd4	Mtnd4
tr	Q8R2K3	Q8R2K3_MOUSE	Ssbp1 mCG_15097	Ssbp1
tr	Q14BI5	Q14BI5_MOUSE	Myom2	Myom2
sp	P24549	AL1A1_MOUSE	1a1 Ahd-2 Ahd2 A	Aldh1a1
tr	B1AR28	B1AR28_MOUSE	Acadvl	Acadvl
sp	P53986	MOT1_MOUSE	Slc16a1 Mct1	Slc16a1
sp	P60867	RS20_MOUSE	Rps20	Rps20
tr	B1AXW6	B1AXW6_MOUSE	Prdx1	Prdx1
sp	Q8BGH2	SAM50_MOUSE	Samm50	Samm50
sp	Q9JHU4	DYHC1_MOUSE	1 Dhc1 Dnch1 Dnch	Dync1h1
sp	P97927	LAMA4_MOUSE	Lama4	Lama4
sp	O70433	FHL2_MOUSE	Fhl2	Fhl2
sp	P42125	ECI1_MOUSE	Eci1 Dci	Eci1
sp	Q3TXS7	PSMD1_MOUSE	Psmd1	Psmd1
sp	Q8VCT4	CES1D_MOUSE	Ces1d Ces1 Ces3	Ces1d
tr	G3UWC2	G3UWC2_MOUSE	Naalad2 mCG_4774	Naalad2
sp	P14152	MDHC_MOUSE	Mdh1 Mor2	Mdh1
sp	Q9CQH7	BT3L4_MOUSE	Btf3l4	Btf3l4
sp	P82349	SGCB_MOUSE	Sgcb	Sgcb
sp	Q9D7N9	APMAP_MOUSE	Apmap	Apmap
tr	A0A0A6YWK5	A0A0A6YWK5_MOU	Ighv1-31	Ighv1-31
tr	Q3UN88	Q3UN88_MOUSE	Mcpt4 mCG_13083	Mcpt4
sp	Q9CPQ8	ATP5L_MOUSE	Atp5l	Atp5l
sp	Q8BFZ9	ERLN2_MOUSE	Erlin2 Spfh2	Erlin2

sp	Q91WS0	CISD1_MOUSE	d1 D10Ertd214e Z(	Cisd1
sp	Q6IFX2	K1C42_MOUSE	Krt42 Ka22	Krt42
sp	O35955	PSB10_MOUSE	smb10 Lmp10 Mec	Psmb10
sp	O88441	MTX2_MOUSE	Mtx2 MNCb-0780	Mtx2
tr	F8WIE1	F8WIE1_MOUSE	Man2c1	Man2c1
sp	P59999	ARPC4_MOUSE	Arpc4 Arc20	Arpc4
sp	P63101	1433Z_MOUSE	Ywhaz	Ywhaz
sp	Q9CQR2	RS21_MOUSE	Rps21	Rps21
sp	Q02257	PLAK_MOUSE	Jup	Jup
tr	F6Y6V5	F6Y6V5_MOUSE	Ndufb5	Ndufb5
sp	Q9JJI8	RL38_MOUSE	Rpl38	Rpl38
sp	Q91YP0	L2HDH_MOUSE	L2hgdh	L2hgdh
sp	Q8BWT1	THIM_MOUSE	Acaa2	Acaa2
sp	Q8QZT1	THIL_MOUSE	Acat1	Acat1
sp	Q9CPR5	RM15_MOUSE	Mrpl15	Mrpl15
sp	P58021	TM9S2_MOUSE	Tm9sf2	Tm9sf2
sp	Q07113	MPRI_MOUSE	Igf2r	Igf2r
sp	Q8VDM4	PSMD2_MOUSE	Psmd2	Psmd2
sp	Q9D0F3	LMAN1_MOUSE	Lman1 Ergic53	Lman1
tr	F6VY18	F6VY18_MOUSE	Gmpr	Gmpr
tr	Q3UI33	Q3UI33_MOUSE	Metap2 METAP2	Metap2
tr	A0A0B4J1H6	0A0B4J1H6_MOUS	Igkv2-137	Igkv2-137
sp	Q8BT60	CPNE3_MOUSE	Cpne3 Kiaa0636	Cpne3
sp	P27467	WNT3A_MOUSE	Wnt3a Wnt-3a	Wnt3a
sp	Q00519	XDH_MOUSE	Xdh	Xdh
sp	P00397	COX1_MOUSE	Mtco1 COI mt-Co1	Mtco1
tr	H3BJ97	H3BJ97_MOUSE	Tinagl1	Tinagl1
sp	Q9DC70	NDUS7_MOUSE	Ndufs7	Ndufs7
sp	Q9D8P4	RM17_MOUSE	Mrpl17	Mrpl17
sp	Q9CQC7	NDUB4_MOUSE	Ndufb4	Ndufb4
sp	Q91W97	HKDC1_MOUSE	Hkdc1	Hkdc1
sp	P03888	NU1M_MOUSE	Mtnd1 mt-Nd1 Nd1	Mtnd1
tr	Q6ZWQ9	Q6ZWQ9_MOUSE	2900073G15Rik m(	Myl12a
tr	Q6P3F7	Q6P3F7_MOUSE	Popdc2	Popdc2
tr	Q642K5	Q642K5_MOUSE	Gm9843 mCG_11	Fau
tr	F6S4G2	F6S4G2_MOUSE	Cds2	Cds2
tr	A2A547	A2A547_MOUSE	Rpl19	Rpl19
tr	A0A140LHC6	0A140LHC6_MOUS	Csrp3	Csrp3
tr	A0A0N4SVQ1	)A0N4SVQ1_MOU	Ndufa4	Ndufa4
sp	Q9D6J5	NDUB8_MOUSE	Ndufb8	Ndufb8
sp	Q9D023	MPC2_MOUSE	Mpc2 Brp44	Mpc2
sp	Q9CZB0	C560_MOUSE	Sdhc	Sdhc
sp	P62267	RS23_MOUSE	Rps23	Rps23
sp	P61514	RL37A_MOUSE	Rpl37a	Rpl37a
sp	P35486	ODPA_MOUSE	Pdha1 Pdha-1	Pdha1
sp	O55100	SNG1_MOUSE	Syngr1	Syngr1
sp	Q8BH59	CMC1_MOUSE	Slc25a12 Aralar1	Slc25a12
tr	A0A1B0GSI7	0A1B0GSI7_MOUS	Col4a1	Col4a1
tr	G3UYV7	G3UYV7_MOUSE	Rps28	Rps28
tr	A0A0R4J275	0A0R4J275_MOUS	dufa12 mCG_112(	Ndufa12

tr	F8WIJ0	F8WIJ0_MOUSE	Slc12a4	Slc12a4
tr	A0A0U1RP13	)A0U1RP13_MOU:	Cdipt	Cdipt
tr	A0A0R4J1Z3	0A0R4J1Z3_MOUS	Tmem33	Tmem33
sp	Q9D898	ARP5L_MOUSE	Arpc5l	Arpc5l
tr	E9Q800	E9Q800_MOUSE	Immt	Immt
sp	Q9CQN7	RM41_MOUSE	Mrpl41	Mrpl41
sp	Q91V79	FITM1_MOUSE	Fitm1 Fit1	Fitm1
sp	Q8C165	P20D1_MOUSE	Pm20d1	Pm20d1
sp	P49817	CAV1_MOUSE	Cav1 Cav	Cav1
sp	Q9DCU6	RM04_MOUSE	Mrpl4 MNCb-3848	Mrpl4
sp	Q91VJ2	CAVN3_MOUSE	:avin3 Prkcdbp Srb	Cavin3
sp	P68372	TBB4B_MOUSE	Tubb4b Tubb2c	Tubb4b
sp	Q8BH95	ECHM_MOUSE	Echs1	Echs1
sp	Q8BH61	F13A_MOUSE	F13a1 F13a	F13a1
tr	Q8C2Q8	Q8C2Q8_MOUSE	Atp5c1	Atp5c1
sp	P42669	PURA_MOUSE	Pura	Pura
sp	P51863	VA0D1_MOUSE	Atp6v0d1 Atp6d	Atp6v0d1
sp	P80314	TCPB_MOUSE	Cct2 Cctb	Cct2
tr	Z4YJF5	Z4YJF5_MOUSE	Myom1	Myom1
tr	E9Q9M1	E9Q9M1_MOUSE	Nt5c2	Nt5c2
tr	G5E8T0	G5E8T0_MOUSE	)najc5 mCG_2306(	Dnajc5
sp	Q9DBS1	TMM43_MOUSE	Tmem43	Tmem43
tr	E9Q7L0	E9Q7L0_MOUSE	Ogdhl	Ogdhl
sp	P38647	GRP75_MOUSE	ı9 Grp75 Hsp74 Hs	Hspa9
tr	E9QKR0	E9QKR0_MOUSE	Gnb2	Gnb2
sp	P03930	ATP8_MOUSE	Itatp8 Atp8 mt-Atp	Mtatp8
tr	A2AI91	A2AI91_MOUSE	Phka1	Phka1
sp	Q9ES83	POPD1_MOUSE	Bves Pop1 Popdc1	Bves
sp	Q921S7	RM37_MOUSE	Mrpl37	Mrpl37
sp	P82348	SGCG_MOUSE	Sgcg	Sgcg
sp	P09411	PGK1_MOUSE	Pgk1 Pgk-1	Pgk1
sp	Q9CQ69	QCR8_MOUSE	Uqcrq	Uqcrq
tr	F8WHP8	F8WHP8_MOUSE	Atp5j2	Atp5j2
sp	Q99M87	DNJA3_MOUSE	Dnaja3 Tid1	Dnaja3
sp	O35295	PURB_MOUSE	Purb	Purb
sp	Q9WVL3	S12A7_MOUSE	Slc12a7 Kcc4	Slc12a7
sp	P83882	RL36A_MOUSE	Rpl36a Rpl44	Rpl36a
sp	Q9CQL5	RM18_MOUSE	Mrpl18	Mrpl18
sp	P06342	HB2Q_MOUSE	H2-Ab1	H2-Ab1
sp	Q61171	PRDX2_MOUSE	Prdx2 Tdpx1 Tpx	Prdx2
sp	Q9CQX2	CYB5B_MOUSE	Cyb5b Cyb5m	Cyb5b
sp	Q9JHZ2	ANKH_MOUSE	Ankh Ank	Ankh
tr	Z4YJR1	Z4YJR1_MOUSE	Lmf1	Lmf1
tr	D3YX76	D3YX76_MOUSE	Gstm2	Gstm2
sp	P17427	AP2A2_MOUSE	Ap2a2 Adtab	Ap2a2
sp	P05132	KAPCA_MOUSE	Prkaca Pkaca	Prkaca
tr	A0A0A6YXW6	)A0A6YXW6_MOU	Igha	Igha
sp	P60202	MYPR_MOUSE	Plp1 Plp	Plp1
sp	P05201	AATC_MOUSE	Got1	Got1
sp	O70423	AOC3_MOUSE	Aoc3 Vap1	Aoc3

sp	P70404	IDHG1_MOUSE	Idh3g	Idh3g
sp	Q8VHX6	FLNC_MOUSE	Flnc Abpl Fln2	Flnc
tr	F6V084	F6V084_MOUSE	Tmx1	Tmx1
tr	A0A0R4J0L6	0A0R4J0L6_MOUS	Mrps35	Mrps35
tr	K3W4T3	K3W4T3_MOUSE	Atp6v0a1	Atp6v0a1
sp	P97499	TEP1_MOUSE	Tep1 Tp1	Tep1
sp	Q8BU88	RM22_MOUSE	Mrpl22	Mrpl22
tr	E9QL80	E9QL80_MOUSE	Lpgat1	Lpgat1
sp	Q9ERI6	RDH14_MOUSE	Rdh14	Rdh14
sp	Q99JI4	PSMD6_MOUSE	Psmd6	Psmd6
tr	F6QKK2	F6QKK2_MOUSE	Arl8a	Arl8a
tr	Q6PHC1	Q6PHC1_MOUSE	Eno1	Eno1
tr	F8WIS9	F8WIS9_MOUSE	Camk2a	Camk2a
sp	P11983	TCPA_MOUSE	Tcp1 Cct1 Ccta	Tcp1
sp	Q9CVB6	ARPC2_MOUSE	Arpc2	Arpc2
sp	P97742	CPT1A_MOUSE	Cpt1a Cpt-1 Cpt1	Cpt1a
tr	H3BKR2	H3BKR2_MOUSE	Gnb1	Gnb1
sp	Q4ZJN1	C1QT9_MOUSE	C1qtnf9	C1qtnf9
tr	E9Q3T0	E9Q3T0_MOUSE	Gm10073	Gm10073
tr	Q7TSG6	Q7TSG6_MOUSE	Rdx	Rdx
tr	D6RH49	D6RH49_MOUSE	Rps27l	Rps27l
sp	O35593	PSDE_MOUSE	Psmd14 Pad1	Psmd14
sp	Q8BY89	CTL2_MOUSE	Slc44a2 Ctl2	Slc44a2
tr	A0A0A6YVS2	0A0A6YVS2_MOUS	Tmco1	Tmco1
sp	Q9CRD2	EMC2_MOUSE	mc2 Kiaa0103 Ttc3	Emc2
tr	A2AP78	A2AP78_MOUSE	Hmgb3	Hmgb3
tr	G3UZT2	G3UZT2_MOUSE	Selenbp2	Selenbp2
sp	P24369	PPIB_MOUSE	Ppib	Ppib
tr	B1AT36	B1AT36_MOUSE	Psmd12	Psmd12
sp	Q9EQ06	DHB11_MOUSE	d17b11 Dhrs8 Pan	Hsd17b11
tr	Q8BHF5	Q8BHF5_MOUSE	Rtn4	Rtn4
sp	Q9EP69	SAC1_MOUSE	acm1l Kiaa0851 Sa	Sacm1l
sp	P97449	AMPN_MOUSE	Anpep Lap-1 Lap1	Anpep
tr	F6SFF5	F6SFF5_MOUSE	Coq9	Coq9
sp	Q3ULD5	MCCB_MOUSE	Mccc2	Mccc2
sp	P61089	UBE2N_MOUSE	Ube2n Blu	Ube2n
sp	Q9CR68	UCRI_MOUSE	Uqcrfs1	Uqcrfs1
tr	H3BKF4	H3BKF4_MOUSE	Gm20708	Gm20708
tr	Q5SW88	Q5SW88_MOUSE	Rab1a Rab1	Rab1a
tr	G5E8R7	G5E8R7_MOUSE	Smyd1	Smyd1
tr	A0A1W2P8E1	0A1W2P8E1_MOU	Ilvbl	Ilvbl
sp	Q6PD26	PIGS_MOUSE	Pigs	Pigs
tr	A0A1L1STE6	0A1L1STE6_MOUS	Idh3a	Idh3a
sp	O55143	AT2A2_MOUSE	Atp2a2	Atp2a2
sp	Q9QWL7	K1C17_MOUSE	Krt17 Krt1-17	Krt17
sp	Q4VAE3	TMM65_MOUSE	Tmem65	Tmem65
sp	Q62165	DAG1_MOUSE	Dag1 Dag-1	Dag1
sp	P19785	ESR1_MOUSE	1 Esr Estr Estra Nr3	Esr1
tr	Q3UAM9	Q3UAM9_MOUSE	Eng	Eng
tr	G3X942	G3X942_MOUSE	alnt9 mCG_12954	Galnt9

tr	G3UZX4	G3UZX4_MOUSE	Csnk2b	Csnk2b
tr	F8WHM5	F8WHM5_MOUSE	Glg1	Glg1
tr	E9PXM6	E9PXM6_MOUSE	Slc29a1	Slc29a1
tr	E9PUE8	E9PUE8_MOUSE	Abcc9	Abcc9
tr	D3Z456	D3Z456_MOUSE	Mrpl3 mCG_14748	Mrpl3
tr	A0A0R4J2A0	0A0R4J2A0_MOUS	Ssh2	Ssh2
sp	Q9WTR5	CAD13_MOUSE	Cdh13	Cdh13
sp	Q8BXV2	BRI3B_MOUSE	Bri3bp	Bri3bp
sp	P35762	CD81_MOUSE	Cd81 Tapa1	Cd81
sp	O54962	BAF_MOUSE	anf1 Baf Bcrp1 L2b	Banf1
tr	Z4YN97	Z4YN97_MOUSE	Ak1	Ak1
tr	Q9D8L3	Q9D8L3_MOUSE	Ssr4 mCG_8079	Ssr4
tr	Q5D073	Q5D073_MOUSE	xmp2 mCG_13421	Pxmp2
tr	F6XWB2	F6XWB2_MOUSE	Igkv1-99 Igkv1-115	Igkv1-99
tr	F6XCE3	F6XCE3_MOUSE	Myl2	Myl2
tr	D3Z198	D3Z198_MOUSE	Mrps17	Mrps17
tr	D3YY42	D3YY42_MOUSE	Yif1b	Yif1b
tr	A2AMH5	A2AMH5_MOUSE	Slc44a1	Slc44a1
tr	A0A1L1SSA8	0A1L1SSA8_MOUS	Tmem205	Tmem205
tr	A0A140LIU4	0A140LIU4_MOUS	ox7a1 mCG_2156	Cox7a1
tr	A0A0U1RNP6	)A0U1RNP6_MOU	Tdrd12	Tdrd12
sp	Q9Z2I9	SUCB1_MOUSE	Sucla2	Sucla2
sp	Q9QZQ8	H2AY_MOUSE	H2afy	H2afy
sp	Q9EP72	EMC7_MOUSE	Emc7 Orf3	Emc7
sp	Q9D6J6	NDUV2_MOUSE	Ndufv2	Ndufv2
sp	Q9D3D9	ATPD_MOUSE	Atp5f1d Atp5d	Atp5f1d
sp	Q9CQZ6	NDUB3_MOUSE	Ndufb3	Ndufb3
sp	Q9CQN6	TM14C_MOUSE	Tmem14c	Tmem14c
sp	Q9CQ40	RM49_MOUSE	Mrpl49	Mrpl49
sp	Q99N92	RM27_MOUSE	Mrpl27	Mrpl27
sp	Q99KP6	PRP19_MOUSE	Prpf19 Prp19 Snev	Prpf19
sp	Q99KI3	EMC3_MOUSE	Emc3 Tmem111	Emc3
sp	Q78IK2	USMG5_MOUSE	Usmg5 Dapit	Usmg5
sp	Q61735	CD47_MOUSE	Cd47	Cd47
sp	Q08AU7	MADL2_MOUSE	Myadml2	Myadml2
sp	P62892	RL39_MOUSE	Rpl39	Rpl39
sp	P62274	RS29_MOUSE	Rps29	Rps29
sp	P57716	NICA_MOUSE	Ncstn	Ncstn
sp	P50247	SAHH_MOUSE	Ahcy	Ahcy
sp	P12815	PDCD6_MOUSE	Pdcd6 Alg2	Pdcd6
sp	P01887	B2MG_MOUSE	B2m	B2m
tr	A2AEY2	A2AEY2_MOUSE	Fhl1 mCG_9696	Fhl1
sp	Q99M71	EPDR1_MOUSE	r1 Epdr2 Merp1 M	Epdr1
sp	Q9JJW5	MYOZ2_MOUSE	Myoz2	Myoz2
sp	Q9EPL8	IPO7_MOUSE	Ipo7 Ranbp7	Ipo7
sp	P61014	PPLA_MOUSE	Pln	Pln
tr	Q3UN10	Q3UN10_MOUSE	Wfs1	Wfs1
tr	B1B1D8	B1B1D8_MOUSE	Mrpl2	Mrpl2
sp	Q91V01	MBOA5_MOUSE	:3 Grcc3f Mboat5 (	Lpcat3
sp	P62259	1433E_MOUSE	Ywhae	Ywhae

tr	B1ARW4	B1ARW4_MOUSE	Ndufs5	Ndufs5
sp	P68373	TBA1C_MOUSE	Tuba1c Tuba6	Tuba1c
sp	Q8VDT9	RM50_MOUSE	Mrpl50	Mrpl50
sp	Q9QWK4	CD5L_MOUSE	Cd5l Aim Api6	Cd5l
sp	P17047	LAMP2_MOUSE	Lamp2 Lamp-2	Lamp2
sp	Q6P3A8	ODBB_MOUSE	Bckdhb	Bckdhb
tr	Q3TRE0	Q3TRE0_MOUSE	Sspn mCG_15024	Sspn
sp	Q9CPU2	NDUB2_MOUSE	Ndufb2	Ndufb2
sp	O08749	DLDH_MOUSE	Dld	Dld
sp	P60229	EIF3E_MOUSE	Eif3e Eif3s6 Int6	Eif3e
sp	Q14C51	PTCD3_MOUSE	Ptcd3 Mrps39	Ptcd3
sp	P97370	AT1B3_MOUSE	Atp1b3	Atp1b3
sp	Q8R429	AT2A1_MOUSE	Atp2a1	Atp2a1
sp	Q9CX56	PSMD8_MOUSE	Psmd8	Psmd8
tr	E9PY26	E9PY26_MOUSE	Blmh	Blmh
sp	P11531	DMD_MOUSE	Dmd	Dmd
tr	D3YVV1	D3YVV1_MOUSE	Tmem30a	Tmem30a
tr	G3UZG6	G3UZG6_MOUSE	Cyb5r1 mCG_5352	Cyb5r1
tr	Q5RL57	Q5RL57_MOUSE	Akap8l mCG_1425	Akap8l
sp	Q9CQ75	NDUA2_MOUSE	Ndufa2	Ndufa2
tr	A0A0G2JGE1	A0A0G2JGE1_MOU	Kyat3	Kyat3
sp	P24668	MPRD_MOUSE	M6pr 46mpr	M6pr
sp	P21981	TGM2_MOUSE	Tgm2	Tgm2
sp	P82198	BGH3_MOUSE	Tgfbi	Tgfbi
tr	G3X8U7	G3X8U7_MOUSE	Ppp3cb mCG_593	Ppp3cb
sp	Q8BFZ3	ACTBL_MOUSE	Actbl2	Actbl2

Significantly differentially enriched proteins (391) in preparations from **β**-cat**Δ**ex3 heart

Gene names	Significant	Length	Enrichment log ratio (beta/wt)	Accession	ID
Pdhx	+	501	4.1098	Q8BKZ9	ODPX_MOUSE
Dlat	+	642	4.1029	Q8BMF4	ODP2_MOUSE
Coq3	+	370	4.0101	Q8BMS4	COQ3_MOUSE
Coq6	+	476	3.9902	Q8R1S0	COQ6_MOUSE
Ogdhl	+	1029	3.6329	E9Q7L0	E9Q7L0_MOUSE
Cpt2 Cpt-2	+	658	3.6044	P52825	CPT2_MOUSE
Vcp	+	806	3.4740	Q01853	TERA_MOUSE
Mcpt4 mCG_130832	+	246	3.4598	Q3UN88	Q3UN88_MOUSE
Ogdh	+	1019	3.2651	Z4YJV4	Z4YJV4_MOUSE
Bckdhb	+	390	3.2128	Q6P3A8	ODBB_MOUSE
Aldh2 Ahd-1 Ahd1	+	519	3.0859	P47738	ALDH2_MOUSE
Galnt9 mCG_129543	+	604	2.9584	G3X942	G3X942_MOUSE
Nid1 Ent	+	1245	2.9551	P10493	NID1_MOUSE
›smb10 Lmp10 Mecl1	+	273	2.9345	O35955	PSB10_MOUSE
Ech1	+	327	2.9164	O35459	ECH1_MOUSE
Psmb6 Lmp19	+	238	2.9118	Q60692	PSB6_MOUSE
Lamb1	+	1834	2.9068	E9QN70	E9QN70_MOUSE
Blmh	+	179	2.9050	E9PY26	E9PY26_MOUSE
Psma4	+	261	2.8703	Q9R1P0	PSA4_MOUSE
Lap3 Lapep	+	519	2.8615	Q9CPY7	AMPL_MOUSE
L2hgdh	+	464	2.8380	Q91YP0	L2HDH_MOUSE
Csrp3	+	65	2.8174	A0A140LHC6	\0A140LHC6_MOUSE
Igkv1-99 Igkv1-115	+	120	2.8094	F6XWB2	F6XWB2_MOUSE
Psma2 Lmpc3	+	234	2.8068	P49722	PSA2_MOUSE
H2-Ab1	+	265	2.7746	P06342	HB2Q_MOUSE
l1 Ftl1-ps1 mCG_172	+	183	2.7124	Q9CPX4	Q9CPX4_MOUSE
Ssh2	+	1429	2.7022	A0A0R4J2A0	\0A0R4J2A0_MOUSE
Psma1	+	263	2.6714	Q9R1P4	PSA1_MOUSE
Ssbp1 mCG_15097	+	148	2.6116	Q8R2K3	Q8R2K3_MOUSE
Psmb7 Mmc14	+	277	2.5977	P70195	PSB7_MOUSE
Psma6	+	246	2.5874	Q9QUM9	PSA6_MOUSE
Psmb3	+	205	2.5855	Q9R1P1	PSB3_MOUSE
Psmb1	+	240	2.5850	O09061	PSB1_MOUSE
Aoc3 Vap1	+	765	2.5846	O70423	AOC3_MOUSE
Psma7	+	248	2.5815	Q9Z2U0	PSA7_MOUSE
Psma3	+	255	2.5680	O70435	PSA3_MOUSE
Ighv1-31	+	117	2.5411	A0A0A6YWK5	.0A0A6YWK5_MOUSE
Lama2	+	3118	2.5382	Q60675	LAMA2_MOUSE
Psmb2	+	201	2.5267	Q9R1P3	PSB2_MOUSE
Lamb2 Lams	+	1799	2.5150	Q61292	LAMB2_MOUSE
.h4f;Hist1h4h;Hist1h4	+	103	2.5078	P62806	H4_MOUSE
Dld	+	509	2.4987	O08749	DLDH_MOUSE
Fth1 Fth	+	182	2.4783	P09528	FRIH_MOUSE
Pfkl Pfk-l Pfkb	+	780	2.4697	P12382	PFKAL_MOUSE
Htra1 Htra Prss11	+	480	2.4690	Q9R118	HTRA1_MOUSE

Echs1	+	290	2.4612	Q8BH95	ECHM_MOUSE
a9 Grp75 Hsp74 Hsp	+	679	2.4076	P38647	GRP75_MOUSE
Psmb5	+	264	2.4587	O55234	PSB5_MOUSE
Psmb4 Lmp3	+	264	2.4053	P99026	PSB4_MOUSE
Glud1 Glud	+	558	2.4026	P26443	DHE3_MOUSE
Kyat3	+	194	2.3956	A0A0G2JGE1	\0A0G2JGE1_MOUSE
Gm3839	+	333	2.3885	S4R1W1	S4R1W1_MOUSE
Dbt	+	482	2.3693	P53395	ODB2_MOUSE
Ighm	+	476	2.3414	A0A075B6A0	\0A075B6A0_MOUSE
Igkv2-137	+	120	2.3358	A0A0B4J1H6	\0A0B4J1H6_MOUSE
H2afy	+	372	2.2989	Q9QZQ8	H2AY_MOUSE
Psma5	+	241	2.2986	Q9Z2U1	PSA5_MOUSE
Pdha1 Pdha-1	+	390	2.2878	P35486	ODPA_MOUSE
Ckm Ckmm	+	381	2.2758	P07310	KCRM_MOUSE
Lactb Lact1	+	551	2.2618	Q9EP89	LACTB_MOUSE
H3f3a	+	119	2.2607	E0CZ27	E0CZ27_MOUSE
1 Dhc1 Dnch1 Dnchc	+	4644	2.2542	Q9JHU4	DYHC1_MOUSE
Acadl	+	430	2.2293	A0A0R4J083	\0A0R4J083_MOUSE
Idh3a	+	384	2.2158	A0A1L1STE6	\0A1L1STE6_MOUSE
Lamc1	+	1607	2.2134	F8VQJ3	F8VQJ3_MOUSE
Tinagl1	+	435	2.2120	H3BJ97	H3BJ97_MOUSE
Psmb8 Lmp7 Mc13	+	276	2.1807	P28063	PSB8_MOUSE
Lama5	+	3718	2.1574	Q61001	LAMA5_MOUSE
Macrod1 Lrp16	+	323	2.1573	Q922B1	MACD1_MOUSE
Sucla2	+	463	2.1059	Q9Z2I9	SUCB1_MOUSE
sr1 Esr Estr Estra Nr3a	+	599	2.0986	P19785	ESR1_MOUSE
Psmd6	+	389	2.0934	Q99JI4	PSMD6_MOUSE
dha Ldh1 mCG_1993	+	332	2.0891	Q564E2	Q564E2_MOUSE
Aldh5a1	+	523	2.0829	Q8BWF0	SSDH_MOUSE
Gm20390	+	267	2.0697	E9PZF0	E9PZF0_MOUSE
Rtn3	+	964	2.0663	Q9ES97	RTN3_MOUSE
Xdh	+	1335	2.0624	Q00519	XDH_MOUSE
Mybpc3	+	1278	2.0497	Q3UIK0	Q3UIK0_MOUSE
Pzp A2m	+	1495	2.0486	Q61838	PZP_MOUSE
Acadvl	+	634	2.0462	B1AR28	B1AR28_MOUSE
Mrps17	+	87	2.0440	D3Z198	D3Z198_MOUSE
Gpi Gpi1	+	558	2.0281	P06745	G6PI_MOUSE
Lama4	+	1816	1.9965	P97927	LAMA4_MOUSE
Pgk1 Pgk-1	+	417	1.9823	P09411	PGK1_MOUSE
Eci1 Dci	+	289	1.9774	P42125	ECI1_MOUSE
Mb	+	154	1.9504	P04247	MYG_MOUSE
Idh2	+	452	1.9449	P54071	IDHP_MOUSE
Ywhaz	+	245	1.9433	P63101	1433Z_MOUSE
Ldhb Ldh-2 Ldh2	+	334	1.8881	P16125	LDHB_MOUSE
Ak1	+	89	1.8831	Z4YN97	Z4YN97_MOUSE
Prdx5	+	213	1.8771	H3BJQ7	H3BJQ7_MOUSE
Cs	+	464	1.8663	Q9CZU6	CISY_MOUSE
Aco2	+	780	1.8157	Q99KI0	ACON_MOUSE
)ab1 Hsp84 Hsp84-1 I	+	724	1.8088	P11499	HS90B_MOUSE
Plp1 Plp	+	277	1.7777	P60202	MYPR_MOUSE

Banf1 Baf Bcrp1 L2bp	+	89	1.7512	O54962	BAF_MOUSE
Man2c1	+	1037	1.7438	F8WIE1	F8WIE1_MOUSE
Flnc Abpl Fln2	+	2726	1.7174	Q8VHX6	FLNC_MOUSE
Gmpr	+	290	1.6999	F6VY18	F6VY18_MOUSE
Hist2h2ab	+	130	1.6935	Q64522	H2A2B_MOUSE
Idh3g	+	393	1.6857	P70404	IDHG1_MOUSE
Cd5l Aim Api6	+	352	1.6629	Q9QWK4	CD5L_MOUSE
Hadha	+	763	1.6586	Q8BMS1	ECHA_MOUSE
Hadhb	+	475	1.6078	Q99JY0	ECHB_MOUSE
Itga7	+	1140	1.6025	Q3TZS3	Q3TZS3_MOUSE
Ywhae	+	255	1.6021	P62259	1433E_MOUSE
Nipsnap2 Gbas	+	281	1.6013	O55126	NIPS2_MOUSE
Prkaca Pkaca	+	351	1.5883	P05132	KAPCA_MOUSE
Trim72 Mg53	+	477	1.5859	Q1XH17	TRI72_MOUSE
Got2 Got-2	+	430	1.5661	P05202	AATM_MOUSE
Eng	+	652	1.5492	Q3UAM9	Q3UAM9_MOUSE
Nid2	+	1403	1.5491	O88322	NID2_MOUSE
Bag2	+	210	1.5434	Q91YN9	BAG2_MOUSE
Cd47	+	303	1.5417	Q61735	CD47_MOUSE
Vps35 Mem3	+	796	1.5238	Q9EQH3	VPS35_MOUSE
Pfkm Pfk-m Pfka	+	780	1.5211	P47857-3	PFKAM_MOUSE
Eno1	+	366	1.5154	Q6PHC1	Q6PHC1_MOUSE
Smyd1	+	456	1.4917	G5E8R7	G5E8R7_MOUSE
Ipo7 Ranbp7	+	1038	1.4726	Q9EPL8	IPO7_MOUSE
Pcca	+	724	1.4657	Q91ZA3	PCCA_MOUSE
Pygm	+	842	1.4610	Q9WUB3	PYGM_MOUSE
Dnaja3 Tid1	+	480	1.4445	Q99M87	DNJA3_MOUSE
Mccc2	+	563	1.4086	Q3ULD5	MCCB_MOUSE
Tgm2	+	686	1.3987	P21981	TGM2_MOUSE
Pln	+	52	1.3753	P61014	PPLA_MOUSE
Cryab Crya2	+	175	1.3713	P23927	CRYAB_MOUSE
Mrps35	+	320	1.3613	A0A0R4J0L6	A0A0R4J0L6_MOUSE
Cav1 Cav	+	178	1.3580	P49817	CAV1_MOUSE
Ephx1	+	455	1.3552	Q9D379	HYEP_MOUSE
Col4a1	+	1562	1.3487	A0A1B0GSI7	A0A1B0GSI7_MOUSE
Tmem65	+	234	1.3297	Q4VAE3	TMM65_MOUSE
Pkm Pk3 Pkm2 Pykm	+	531	1.3038	P52480-2	KPYM_MOUSE
Uqcrb mCG_67985	+	111	1.3011	Q9CQB4	Q9CQB4_MOUSE
Cat Cas-1 Cas1	+	527	1.2849	P24270	CATA_MOUSE
Aldoa Aldo1	+	364	1.2840	P05064	ALDOA_MOUSE
Pccb	+	504	1.2636	E9Q1J7	E9Q1J7_MOUSE
Gpx4	+	157	1.2296	S4R1E5	S4R1E5_MOUSE
Fhl2	+	279	1.2258	O70433	FHL2_MOUSE
Des	+	469	1.2201	P31001	DESM_MOUSE
Tubb4b Tubb2c	+	445	1.2055	P68372	TBB4B_MOUSE
[illegible] Glna1 Hbb-bt Hbbt1	+	147	1.1963	A8DUK4	A8DUK4_MOUSE
Pygb	+	843	1.1898	Q8CI94	PYGB_MOUSE
Coq9	+	270	1.1641	F6SFF5	F6SFF5_MOUSE
Etfdh	+	616	1.1479	Q921G7	ETFD_MOUSE
Eef1a2 Eef1al Stn	+	463	1.1299	P62631	EF1A2_MOUSE

Hist1h2bp	+	126	1.1270	Q8CGP2	H2B1P_MOUSE
Tdrd12	+	127	1.1188	A0A0U1RNP6	A0A0U1RNP6_MOUSE
Actn2	+	894	1.1159	Q9JI91	ACTN2_MOUSE
Dnajc5 mCG_23060	+	167	1.1154	G5E8T0	G5E8T0_MOUSE
Bdh1 Bdh	+	343	1.1076	Q80XN0	BDH_MOUSE
lh1a1 Ahd-2 Ahd2 Al(	+	501	1.1006	P24549	AL1A1_MOUSE
Cavin2 Sdpr Sdr	+	418	1.0817	Q63918	CAVN2_MOUSE
Cyb5r1 mCG_5352	+	176	1.0773	G3UZG6	G3UZG6_MOUSE
Mgst3	+	153	1.0676	Q9CPU4	MGST3_MOUSE
Naalad2 mCG_4774	+	778	1.0447	G3UWC2	G3UWC2_MOUSE
Ndufa6	+	131	1.0155	Q9CQZ5	NDUA6_MOUSE
Dmd	+	3678	1.0089	P11531	DMD_MOUSE
Hspa8 Hsc70 Hsc73	+	646	1.0088	P63017	HSP7C_MOUSE
Myoz2	+	264	1.0087	Q9JJW5	MYOZ2_MOUSE
Fhl1 mCG_9696	+	323	1.0064	A2AEY2	A2AEY2_MOUSE
Entpd2 Cd39l1	+	495	0.9697	O55026	ENTP2_MOUSE
Prdx1	+	176	0.9655	B1AXW6	B1AXW6_MOUSE
Gys1	+	674	0.9650	A0A1B0GT92	A0A1B0GT92_MOUSE
Samm50	+	469	0.9593	Q8BGH2	SAM50_MOUSE
Tpi1 Tpi	+	299	0.9583	P17751	TPIS_MOUSE
Sdha	+	664	0.9492	Q8K2B3	SDHA_MOUSE
Phb2 Bap Bcap37 Rea	+	299	0.9354	O35129	PHB2_MOUSE
Cavin1 Ptrf	+	392	0.9267	O54724	CAVN1_MOUSE
Itgb1	+	798	0.9229	P09055	ITB1_MOUSE
Ehd2	+	543	0.9182	Q8BH64	EHD2_MOUSE
Gstm2	+	184	0.9170	D3YX76	D3YX76_MOUSE
Ttn	+	35213	0.9112	A2ASS6	TITIN_MOUSE
Ptcd3 Mrps39	+	685	0.9012	Q14C51	PTCD3_MOUSE
Ndufs7	+	224	0.8814	Q9DC70	NDUS7_MOUSE
Ahcy	+	432	0.8622	P50247	SAHH_MOUSE
Rtn4	+	375	0.8585	Q8BHF5	Q8BHF5_MOUSE
globin alpha 1 haema	+	142	0.8533	Q91VB8	Q91VB8_MOUSE
Jup	+	745	0.8501	Q02257	PLAK_MOUSE
Vdac3	+	284	0.8285	J3QMG3	J3QMG3_MOUSE
Mrpl21 D9Wsu149	+	209	0.8160	Q9D1N9	RM21_MOUSE
Ndufs2	+	437	0.8160	D3YXT0	D3YXT0_MOUSE
Vdac1 Vdac5	+	296	0.8078	Q60932	VDAC1_MOUSE
Tuba1c Tuba6	+	449	0.8045	P68373	TBA1C_MOUSE
Ndufa5	+	116	0.7993	Q9CPP6	NDUA5_MOUSE
Reep5 mCG_121499	+	189	0.7866	G3X8R0	G3X8R0_MOUSE
Mrpl13	+	178	0.7861	Q9D1P0	RM13_MOUSE
Mtch2	+	312	0.7831	Q9D050	Q9D050_MOUSE
Csnk2b	+	257	0.7793	G3UZX4	G3UZX4_MOUSE
Dsp	+	2883	0.7608	E9Q557	DESP_MOUSE
Csnk2a1 Ckiia	+	391	0.7459	Q60737	CSK21_MOUSE
Tgfbi	+	683	0.7401	P82198	BGH3_MOUSE
isd1 D10Ertd214e Zc(	+	108	0.7379	Q91WS0	CISD1_MOUSE
Sgca	+	387	0.7277	P82350	SGCA_MOUSE
Ugp2	+	508	0.7158	Q91ZJ5	UGPA_MOUSE
Slc16a1 Mct1	+	493	0.7145	P53986	MOT1_MOUSE

Ptgfrn Fprp	+	879	0.7145	Q9WV91	FPRP_MOUSE
Ndufa9	+	373	0.7120	A0A0R3P9C8	\0A0R3P9C8_MOUSE
Stom Epb7.2 Epb72	+	284	0.6857	P54116	STOM_MOUSE
Mtx2 MNCb-0780	+	263	0.6442	O88441	MTX2_MOUSE
Slc44a2 Ctl2	+	706	0.5835	Q8BY89	CTL2_MOUSE
Ndufs3	+	263	0.5574	Q9DCT2	NDUS3_MOUSE
dr1 Epdr2 Merp1 Me	+	224	0.5488	Q99M71	EPDR1_MOUSE
Arl8a	+	165	0.5311	F6QKK2	F6QKK2_MOUSE
Alb Alb-1 Alb1	+	608	0.5223	P07724	ALBU_MOUSE
Mdh2 Mor1	+	338	0.5211	P08249	MDHM_MOUSE
Dpep1 Mbd1 Rdp	+	410	0.5190	P31428	DPEP1_MOUSE
acm1l Kiaa0851 Sac	+	587	0.4875	Q9EP69	SAC1_MOUSE
Prdx2 Tdpx1 Tpx	+	198	0.4494	Q61171	PRDX2_MOUSE
ps27a Uba80 Ubcep	+	156	0.4488	P62983	RS27A_MOUSE
Fitm1 Fit1	+	292	0.4422	Q91V79	FITM1_MOUSE
Sgcb	+	320	0.4283	P82349	SGCB_MOUSE
Camk2a	+	489	0.3876	F8WIS9	F8WIS9_MOUSE
Rtn2 Nspl1	+	471	0.3439	O70622	RTN2_MOUSE
Acaa2	+	397	-0.2803	Q8BWT1	THIM_MOUSE
Casq2	+	418	-0.3657	F6QYE1	F6QYE1_MOUSE
Ckmt2	+	419	-0.4170	Q6P8J7	KCRS_MOUSE
Col6a6	+	2265	-0.4329	E9Q6A6	E9Q6A6_MOUSE
Tmx2 Txndc14	+	295	-0.4984	Q9D710	TMX2_MOUSE
Psmd2	+	908	-0.5293	Q8VDM4	PSMD2_MOUSE
Ap2a2 Adtab	+	938	-0.6093	P17427	AP2A2_MOUSE
Ndufa4	+	49	-0.6178	A0A0N4SVQ1	.0A0N4SVQ1_MOUSE
Atp6v0a1	+	838	-0.6295	K3W4T3	K3W4T3_MOUSE
Erlin2 Spfh2	+	340	-0.6311	Q8BFZ9	ERLN2_MOUSE
Uqcrc2	+	453	-0.6351	Q9DB77	QCR2_MOUSE
Atp5c1	+	274	-0.6399	Q8C2Q8	Q8C2Q8_MOUSE
Cct3 Cctg	+	545	-0.6545	P80318	TCPG_MOUSE
Atp2a2	+	1044	-0.6705	O55143	AT2A2_MOUSE
Apmap	+	415	-0.6790	Q9D7N9	APMAP_MOUSE
Cox7a1 mCG_21566	+	89	-0.6813	A0A140LIU4	\0A140LIU4_MOUSE
Slc25a3 mCG_10343	+	358	-0.7164	G5E902	G5E902_MOUSE
Pdcd6 Alg2	+	191	-0.7419	P12815	PDCD6_MOUSE
Gnb2	+	382	-0.7542	E9QKR0	E9QKR0_MOUSE
Tep1 Tp1	+	2629	-0.7824	P97499	TEP1_MOUSE
Atp1a2	+	1020	-0.8155	Q6PIE5	AT1A2_MOUSE
Rpn1	+	608	-0.8393	Q91YQ5	RPN1_MOUSE
Tmed7	+	188	-0.8637	E9Q7G1	E9Q7G1_MOUSE
Tmem38a	+	298	-0.8673	Q3TMP8	TM38A_MOUSE
Ces1d Ces1 Ces3	+	565	-0.8699	Q8VCT4	CES1D_MOUSE
Tm9sf2	+	662	-0.8750	P58021	TM9S2_MOUSE
Cct7	+	502	-0.8783	A0A0N4SV00	\0A0N4SV00_MOUSE
lat1 Alcat1 Gm91 Lyc	+	376	-0.9030	Q3UN02	LCLT1_MOUSE
Lman2	+	358	-0.9481	Q9DBH5	LMAN2_MOUSE
Ddost	+	441	-0.9538	O54734	OST48_MOUSE
Myh13	+	1938	-0.9544	B1AR69	B1AR69_MOUSE
Cacna2d1	+	1091	-0.9766	E9Q1X8	E9Q1X8_MOUSE

Cct8	+	489	-0.9896	H3BL49	H3BL49_MOUSE
Arpc2	+	300	-0.9983	Q9CVB6	ARPC2_MOUSE
Emc3 Tmem111	+	261	-1.0256	Q99KI3	EMC3_MOUSE
Ryr2	+	4966	-1.0510	F6U7V1	F6U7V1_MOUSE
Tcp1 Cct1 Ccta	+	556	-1.0733	P11983	TCPA_MOUSE
Tmed4	+	170	-1.0835	Q5SVW9	Q5SVW9_MOUSE
Emc1 Kiaa0090	+	997	-1.0915	Q8C7X2	EMC1_MOUSE
Atp5o D12Wsu28e	+	213	-1.0982	Q9DB20	ATPO_MOUSE
Hist1h1d H1f3	+	221	-1.0985	P43277	H13_MOUSE
Lmf1	+	574	-1.1219	Z4YJR1	Z4YJR1_MOUSE
Agpat3 Lpaat3	+	376	-1.1243	Q9D517	PLCC_MOUSE
Emc2 Kiaa0103 Ttc35	+	297	-1.1373	Q9CRD2	EMC2_MOUSE
Atp1a1	+	1023	-1.1466	Q8VDN2	AT1A1_MOUSE
ceh1 Aadacl1 Kiaa13(	+	408	-1.1495	Q8BLF1	NCEH1_MOUSE
Slc27a1 Fatp Fatp1	+	646	-1.1702	Q60714	S27A1_MOUSE
Hhatl Gup1 Kiaa1173	+	503	-1.1813	Q9D1G3	HHATL_MOUSE
Cdh13	+	714	-1.1936	Q9WTR5	CAD13_MOUSE
Myh6 Myhca	+	1938	-1.2190	Q02566	MYH6_MOUSE
Emc7 Orf3	+	241	-1.2636	Q9EP72	EMC7_MOUSE
Canx	+	591	-1.2745	P35564	CALX_MOUSE
Cct2 Cctb	+	535	-1.3004	P80314	TCPB_MOUSE
Akap8l mCG_14258	+	641	-1.3108	Q5RL57	Q5RL57_MOUSE
Tmco1	+	200	-1.3311	A0A0A6YVS2	\0A0A6YVS2_MOUSE
Purb	+	324	-1.3690	O35295	PURB_MOUSE
Pigs	+	555	-1.4212	Q6PD26	PIGS_MOUSE
Lnpep	+	1025	-1.4310	Q8C129	LCAP_MOUSE
Pm20d1	+	503	-1.4322	Q8C165	P20D1_MOUSE
Fabp3 Fabph1	+	133	-1.4529	P11404	FABPH_MOUSE
Tmed10 Tmp21	+	219	-1.4536	Q9D1D4	TMEDA_MOUSE
Cct6a Cct6 Cctz Cctz1	+	531	-1.4953	P80317	TCPZ_MOUSE
C1qtnf9	+	333	-1.5074	Q4ZJN1	C1QT9_MOUSE
Btf3l4	+	158	-1.6042	Q9CQH7	BT3L4_MOUSE
Arpc4 Arc20	+	168	-1.6426	P59999	ARPC4_MOUSE
Bri3bp	+	253	-1.6478	Q8BXV2	BRI3B_MOUSE
Atp5f1a Atp5a1	+	553	-1.6719	Q03265	ATPA_MOUSE
Rpl28	+	137	-1.7194	P41105	RL28_MOUSE
tco2 COII COX2 mt-C(	+	227	-1.8520	P00405	COX2_MOUSE
Hspa5 Grp78	+	655	-1.8558	P20029	GRP78_MOUSE
Aspn	+	373	-1.8669	Q99MQ4	ASPN_MOUSE
Pacsin3	+	424	-1.9030	Q99JB8	PACN3_MOUSE
Capza2 Cappa2	+	286	-1.9515	P47754	CAZA2_MOUSE
Arpc5l	+	153	-1.9917	Q9D898	ARP5L_MOUSE
Atp6v0d1 Atp6d	+	351	-2.0168	P51863	VA0D1_MOUSE
Rps15	+	118	-2.0568	D3YTQ9	D3YTQ9_MOUSE
Actbl2	+	376	-2.0846	Q8BFZ3	ACTBL_MOUSE
Phkb	+	1085	-2.1200	Q7TSH2	KPBB_MOUSE
Naca	+	215	-2.1234	Q60817	NACA_MOUSE
Pura	+	321	-2.1381	P42669	PURA_MOUSE
Parp4	+	1969	-2.1428	E9PYK3	E9PYK3_MOUSE
Phka1	+	1182	-2.1645	A2AI91	A2AI91_MOUSE

Tnnc1	+	106	-2.1664	E9Q8P0	E9Q8P0_MOUSE
Ilvbl	+	596	-2.1796	A0A1W2P8E1	.0A1W2P8E1_MOUSE
Actr1a Ctrn1	+	376	-2.2263	P61164	ACTZ_MOUSE
Myl3 Mlc1v Mylc	+	204	-2.2313	P09542	MYL3_MOUSE
Prps1l3	+	318	-2.2371	G3UXL2	G3UXL2_MOUSE
Srl Sar	+	910	-2.2762	Q7TQ48	SRCA_MOUSE
Myl6	+	158	-2.2796	A0A1W2P7Q9	0A1W2P7Q9_MOUSE
Rps12 mCG_132913	+	132	-2.3082	Q6ZWZ6	Q6ZWZ6_MOUSE
Myh9	+	1960	-2.3159	Q8VDD5	MYH9_MOUSE
Dnpep	+	475	-2.4278	Q3TVK3	Q3TVK3_MOUSE
Tfrc Trfr	+	763	-2.4463	Q62351	TFR1_MOUSE
Myh14	+	2000	-2.4666	Q6URW6-3	MYH14_MOUSE
Rpl26	+	145	-2.4864	P61255	RL26_MOUSE
Btf3	+	204	-2.5145	Q64152	BTF3_MOUSE
Tnni3	+	211	-2.5764	P48787	TNNI3_MOUSE
Uqcrfs1	+	274	-2.6115	Q9CR68	UCRI_MOUSE
Myh11	+	1938	-2.6128	E9QPE7	E9QPE7_MOUSE
Lman1 Ergic53	+	517	-2.6782	Q9D0F3	LMAN1_MOUSE
Tpp2	+	1262	-2.6937	Q64514	TPP2_MOUSE
Rps26	+	115	-2.7659	P62855	RS26_MOUSE
Rpl30	+	115	-2.7942	P62889	RL30_MOUSE
Capzb Cappb1	+	277	-2.8065	P47757	CAPZB_MOUSE
Rpl37a	+	92	-2.8084	P61514	RL37A_MOUSE
Atp5f1d Atp5d	+	168	-2.9297	Q9D3D9	ATPD_MOUSE
Nnt	+	1086	-2.9453	Q61941	NNTM_MOUSE
ι 2900073G15Rik mC(	+	172	-2.9549	Q6ZWQ9	Q6ZWQ9_MOUSE
Rps9	+	194	-2.9864	Q6ZWN5	RS9_MOUSE
ack1 Gnb2-rs1 Gnb2l	+	317	-3.2095	P68040	RACK1_MOUSE
Rpl36a Rpl44	+	106	-3.2234	P83882	RL36A_MOUSE
Actg2 Acta3 Actsg	+	376	-3.3905	P63268	ACTH_MOUSE
Rpl18a	+	147	-3.4019	A0A1D5RLW5	.0A1D5RLW5_MOUSE
Gm10073	+	114	-3.5222	E9Q3T0	E9Q3T0_MOUSE
Rps17	+	135	-3.5435	P63276	RS17_MOUSE
Atp5f1b Atp5b	+	529	-3.5537	P56480	ATPB_MOUSE
Rps27	+	83	-3.5728	A0A0G2JG29	A0A0G2JG29_MOUSE
Rpl3	+	403	-3.6222	P27659	RL3_MOUSE
Rps4x Rps4	+	263	-3.6711	P62702	RS4X_MOUSE
mCG_121646 mCG_	+	160	-3.7029	Q9CQM8	Q9CQM8_MOUSE
Rpl3l	+	407	-3.7065	E9PWZ3	E9PWZ3_MOUSE
Rps2 Llrep3 Rps4	+	293	-3.7486	P25444	RS2_MOUSE
	+	NaN	-3.7738	E9QAZ2	E9QAZ2_MOUSE
Rps15a	+	130	-3.7741	P62245	RS15A_MOUSE
Rpl32	+	135	-3.7987	P62911	RL32_MOUSE
Rpl5	+	297	-3.8025	P47962	RL5_MOUSE
Acta1 Acta	+	377	-3.8833	P68134	ACTS_MOUSE
Rps27l	+	77	-3.9415	D6RH49	D6RH49_MOUSE
Rpl35	+	123	-4.1435	Q6ZWV7	RL35_MOUSE
Tpm1 Tpm-1 Tpma	+	284	-4.1631	P58771	TPM1_MOUSE
Rps24	+	118	-4.1738	A0A286YEB7	A0A286YEB7_MOUSE
Rps23	+	143	-4.1813	P62267	RS23_MOUSE

Rps29	+	56	-4.1865	P62274	RS29_MOUSE
Rpl24	+	157	-4.2035	Q8BP67	RL24_MOUSE
Tnnt2	+	302	-4.2333	Q6P3Z7	Q6P3Z7_MOUSE
Metap2 METAP2	+	488	-4.2694	Q3UI33	Q3UI33_MOUSE
Rps11	+	158	-4.2707	P62281	RS11_MOUSE
Rpl7	+	270	-4.2774	P14148	RL7_MOUSE
Rpl38	+	70	-4.2821	Q9JJI8	RL38_MOUSE
Rpl6	+	296	-4.2898	P47911	RL6_MOUSE
Rps3a Rps3a1	+	264	-4.3293	P97351	RS3A_MOUSE
Gm9493	+	192	-4.3976	F6SVV1	F6SVV1_MOUSE
Rps25	+	93	-4.4060	A0A1L1SQA8	\0A1L1SQA8_MOUSE
Rpl22	+	128	-4.4327	P67984	RL22_MOUSE
Rpl27	+	136	-4.4536	P61358	RL27_MOUSE
Rpl39	+	51	-4.4758	P62892	RL39_MOUSE
Rpl8	+	257	-4.4841	P62918	RL8_MOUSE
Rpl36 mCG_20352	+	105	-4.4950	Q6ZWZ4	Q6ZWZ4_MOUSE
u Gm9843 mCG_117	+	133	-4.5989	Q642K5	Q642K5_MOUSE
m11361 mCG_11667	+	152	-4.7011	A0A1Y7VKY1	\0A1Y7VKY1_MOUSE
Rpl7a Surf-3 Surf3	+	266	-4.7201	P12970	RL7A_MOUSE
Rps13 mCG_123365	+	140	-4.7383	Q921R2	Q921R2_MOUSE
ı mCG_123122 mCG_	+	217	-4.8183	Q5XJF6	Q5XJF6_MOUSE
Rps3	+	243	-4.8244	P62908	RS3_MOUSE
Rpl9	+	191	-4.9191	A0A0G2JES3	\0A0G2JES3_MOUSE
Rpl31	+	125	-4.9269	P62900	RL31_MOUSE
Rps19	+	145	-4.9848	Q9CZX8	RS19_MOUSE
Rps20	+	119	-5.0665	P60867	RS20_MOUSE
Rps8	+	208	-5.1370	P62242	RS8_MOUSE
ɔl10 RP23-436K3.4-0(	+	201	-5.1404	I7HLV2	I7HLV2_MOUSE
Rpl12	+	165	-5.1819	P35979	RL12_MOUSE
	+	NaN	-5.1846	A0A140T8M7	\0A140T8M7_MOUSE
Rps14	+	151	-5.2275	P62264	RS14_MOUSE
Rps16	+	146	-5.2992	P14131	RS16_MOUSE
pl13a P198 Tstap198	+	203	-5.3246	P19253	RL13A_MOUSE
Rpl23	+	140	-5.4155	P62830	RL23_MOUSE
Rps5 mCG_22552	+	204	-5.4256	Q91V55	Q91V55_MOUSE
Rpl18	+	159	-5.4547	A0A1B0GQU8	.0A1B0GQU8_MOUSE
Rpsa Lamr1 P40-8	+	295	-5.4677	P14206	RSSA_MOUSE
Rpl4	+	419	-5.5740	Q9D8E6	RL4_MOUSE
Rpl13	+	211	-5.6277	P47963	RL13_MOUSE
Rplp0 Arbp	+	317	-5.6877	P14869	RLA0_MOUSE
Rps10	+	154	-5.7804	Q3UW83	Q3UW83_MOUSE
Rps6	+	249	-5.8448	P62754	RS6_MOUSE
Rpl27a	+	148	-5.8890	P14115	RL27A_MOUSE
Rpl17	+	184	-5.9614	Q9CPR4	RL17_MOUSE
Rpl14	+	217	-6.0021	Q9CR57	RL14_MOUSE
Rps28	+	56	-6.0562	G3UYV7	G3UYV7_MOUSE
T	C	N	N	T	T
Prpf19 Prp19 Snev		504	2.0451	Q99KP6	PRP19_MOUSE
Hspg2		4383	1.5194	E9PZ16	E9PZ16_MOUSE
Hmgb3		159	1.2337	A2AP78	A2AP78_MOUSE

Popdc2	371	1.0543	Q6P3F7	Q6P3F7_MOUSE
Myl2	62	1.0307	F6XCE3	F6XCE3_MOUSE
Sdhb	282	1.0166	Q9CQA3	SDHB_MOUSE
Wnt3a Wnt-3a	352	0.9863	P27467	WNT3A_MOUSE
Got1	413	0.8448	P05201	AATC_MOUSE
Cavin3 Prkcdbp Srbc	260	0.8169	Q91VJ2	CAVN3_MOUSE
Anxa4 Anx4	319	0.7753	P97429	ANXA4_MOUSE
Igha	389	0.7456	A0A0A6YXW6	.0A0A6YXW6_MOUSE
Anpep Lap-1 Lap1	966	0.7312	P97449	AMPN_MOUSE
Vdac2 Vdac6	295	0.6834	Q60930	VDAC2_MOUSE
Ppib	216	0.6607	P24369	PPIB_MOUSE
Dlst	454	0.6574	Q9D2G2	ODO2_MOUSE
Maob	520	0.6500	Q8BW75	AOFB_MOUSE
Mpc2 Brp44	127	0.6287	Q9D023	MPC2_MOUSE
Cpt1a Cpt-1 Cpt1	773	0.6267	P97742	CPT1A_MOUSE
Rac1 mCG_23557	211	0.5938	Q3TLP8	Q3TLP8_MOUSE
Ssr4 mCG_8079	173	0.5603	Q9D8L3	Q9D8L3_MOUSE
Immt	679	0.5533	E9Q800	E9Q800_MOUSE
Ndufa13 Grim19	144	0.5387	Q9ERS2	NDUAD_MOUSE
Igf2r	2483	0.5370	Q07113	MPRI_MOUSE
Sgcg	291	0.5368	P82348	SGCG_MOUSE
Dhrs4 D14Ucla2	279	0.5359	Q99LB2	DHRS4_MOUSE
Mtco1 COI mt-Co1	514	0.5253	P00397	COX1_MOUSE
Usmg5 Dapit	58	0.4902	Q78IK2	USMG5_MOUSE
Phb	272	0.4897	P67778	PHB_MOUSE
Gm20708	155	0.4646	H3BKF4	H3BKF4_MOUSE
Mrpl17	176	0.4631	Q9D8P4	RM17_MOUSE
Mtnd1 mt-Nd1 Nd1	318	0.4601	P03888	NU1M_MOUSE
Eif3e Eif3s6 Int6	445	0.4585	P60229	EIF3E_MOUSE
Ppp3cb mCG_5935	515	0.4565	G3X8U7	G3X8U7_MOUSE
Ndufv1	455	0.4507	D3YUM1	D3YUM1_MOUSE
Slc25a4 Anc1 Ant1	298	0.4467	P48962	ADT1_MOUSE
Myadm	81	0.4328	A0A0N4SW94	.0A0N4SW94_MOUSE
Mrps30	442	0.4219	Q9D0G0	RT30_MOUSE
Atp1b1 Atp4b	304	0.4212	P14094	AT1B1_MOUSE
Hmgb1	211	0.4198	A0A0J9YUZ4	A0A0J9YUZ4_MOUSE
Ndufa2	99	0.4195	Q9CQ75	NDUA2_MOUSE
Mvp	870	0.4119	E9Q3X0	E9Q3X0_MOUSE
Psmd12	436	0.4058	B1AT36	B1AT36_MOUSE
Mrpl22	206	0.4056	Q8BU88	RM22_MOUSE
Cycs	105	0.3948	P62897	CYC_MOUSE
Slc25a5 Ant2	298	0.3875	P51881	ADT2_MOUSE
Cpt1b	772	0.3824	Q924X2	CPT1B_MOUSE
F13a1 F13a	732	0.3770	Q8BH61	F13A_MOUSE
Slc25a13 Aralar2	676	0.3646	Q9QXX4	CMC2_MOUSE
Cox6b1 Cox6b	86	0.3642	P56391	CX6B1_MOUSE
Anxa11 Anx11	503	0.3617	P97384	ANX11_MOUSE
Sccpdh	429	0.3520	Q8R127	SCPDL_MOUSE
Mrpl15	295	0.3413	Q9CPR5	RM15_MOUSE
Atp5l	103	0.3407	Q9CPQ8	ATP5L_MOUSE

Abcc9	1533	0.3384	E9PUE8	E9PUE8_MOUSE
Cyb5r3	313	0.3301	F2Z456	F2Z456_MOUSE
Ube2n Blu	152	0.3224	P61089	UBE2N_MOUSE
Cox5a	146	0.3192	P12787	COX5A_MOUSE
Bcam Gplu Lu	622	0.3172	Q9R069	BCAM_MOUSE
Mtnd4 mt-Nd4 Nd4	459	0.3099	P03911	NU4M_MOUSE
ipoq Acdc Acrp30 Apm1	247	0.3024	Q60994	ADIPO_MOUSE
'smd3 P91a Tstap91a	530	0.2927	P14685	PSMD3_MOUSE
Sgcd	289	0.2869	P82347	SGCD_MOUSE
Rdh14	334	0.2849	Q9ERI6	RDH14_MOUSE
Tmem33	246	0.2800	A0A0R4J1Z3	\0A0R4J1Z3_MOUSE
Atp5j2	76	0.2716	F8WHP8	F8WHP8_MOUSE
Mrpl50	159	0.2703	Q8VDT9	RM50_MOUSE
Ndufb3	104	0.2699	Q9CQZ6	NDUB3_MOUSE
Mrpl18	180	0.2608	Q9CQL5	RM18_MOUSE
Ndufb7	137	0.2484	Q9CR61	NDUB7_MOUSE
tp5i Atp5k Lfm-1 Lfm1	71	0.2455	Q06185	ATP5I_MOUSE
Acsl1	699	0.2452	D3Z041	D3Z041_MOUSE
Ndufs8	212	0.2424	Q8K3J1	NDUS8_MOUSE
Myom1	1569	0.2417	Z4YJF5	Z4YJF5_MOUSE
Rab1a Rab1	202	0.2384	Q5SW88	Q5SW88_MOUSE
Mrpl49	166	0.2329	Q9CQ40	RM49_MOUSE
Sptbn1	2092	0.2278	A0A0A0MQG2	0A0A0MQG2_MOUSE
Slc12a7 Kcc4	1083	0.2227	Q9WVL3	S12A7_MOUSE
Mrpl4 MNCb-3848	294	0.2163	Q9DCU6	RM04_MOUSE
Dhrs7c Sdr32c2	311	0.2072	Q8CHS7	DRS7C_MOUSE
Bves Pop1 Popdc1	358	0.2024	Q9ES83	POPD1_MOUSE
Ndufb4	129	0.2000	Q9CQC7	NDUB4_MOUSE
Psmd14 Pad1	310	0.1932	O35593	PSDE_MOUSE
Cav3	151	0.1895	P51637	CAV3_MOUSE
Ndufa12 mCG_11204	149	0.1892	A0A0R4J275	\0A0R4J275_MOUSE
Cyc1	325	0.1871	Q9D0M3	CY1_MOUSE
Mdh1 Mor2	334	0.1816	P14152	MDHC_MOUSE
Atp5f1	256	0.1784	Q9CQQ7	AT5F1_MOUSE
Atp1b3	278	0.1734	P97370	AT1B3_MOUSE
Slc25a11	314	0.1488	Q9CR62	M2OM_MOUSE
Gnai2 Gnai-2	355	0.1439	P08752	GNAI2_MOUSE
Fbn1 Fbn-1	2873	0.1406	Q61554	FBN1_MOUSE
Krt17 Krt1-17	433	0.1301	Q9QWL7	K1C17_MOUSE
Cd81 Tapa1	236	0.1167	P35762	CD81_MOUSE
Fitm2 Fit2	262	0.1163	P59266	FITM2_MOUSE
Dag1 Dag-1	893	0.1058	Q62165	DAG1_MOUSE
Ndufb10	176	0.1052	Q9DCS9	NDUBA_MOUSE
Myadml2	307	0.1043	Q08AU7	MADL2_MOUSE
Ndufb5	135	0.0946	F6Y6V5	F6Y6V5_MOUSE
Krt42 Ka22	452	0.0738	Q6IFX2	K1C42_MOUSE
Psmd8	353	0.0720	Q9CX56	PSMD8_MOUSE
Psmd1	953	0.0716	Q3TXS7	PSMD1_MOUSE
Wfs1	814	0.0627	Q3UN10	Q3UN10_MOUSE
lsd17b11 Dhrs8 Pan1b	298	0.0531	Q9EQ06	DHB11_MOUSE

Ndufb8	186	0.0481	Q9D6J5	NDUB8_MOUSE
Mrpl41	135	0.0431	Q9CQN7	RM41_MOUSE
Col6a3	2677	0.0419	J3QQ16	J3QQ16_MOUSE
Col6a1	1025	0.0183	Q04857	CO6A1_MOUSE
Sdhc	169	0.0140	Q9CZB0	C560_MOUSE
Col6a2	1034	-0.0081	Q02788	CO6A2_MOUSE
Ndufv2	248	-0.0098	Q9D6J6	NDUV2_MOUSE
Cd36	472	-0.0130	Q08857	CD36_MOUSE
Ndufs6 Ip13	116	-0.0136	P52503	NDUS6_MOUSE
Cox4i1 Cox4 Cox4a	169	-0.0245	P19783	COX41_MOUSE
Cltc	1679	-0.0281	Q5SXR6	Q5SXR6_MOUSE
Atp2a1	994	-0.0410	Q8R429	AT2A1_MOUSE
Atp8a1 Atpc1	1164	-0.0426	P70704	AT8A1_MOUSE
	NaN	-0.0434	G3X9L6	G3X9L6_MOUSE
Slc2a4 Glut-4 Glut4	509	-0.0467	P14142	GTR4_MOUSE
Ankh Ank	492	-0.0545	Q9JHZ2	ANKH_MOUSE
Cyb5b Cyb5m	146	-0.0606	Q9CQX2	CYB5B_MOUSE
B2m	119	-0.0618	P01887	B2MG_MOUSE
Fn1	2361	-0.0711	Q3UHL6	Q3UHL6_MOUSE
Ndufa10	355	-0.0711	Q99LC3	NDUAA_MOUSE
M6pr 46mpr	278	-0.0736	P24668	MPRD_MOUSE
Cct4	509	-0.0754	G5E839	G5E839_MOUSE
Ndufs1	727	-0.0848	Q91VD9	NDUS1_MOUSE
Slc25a12 Aralar1	677	-0.0876	Q8BH59	CMC1_MOUSE
Rdx	389	-0.1033	Q7TSG6	Q7TSG6_MOUSE
Ndufb2	105	-0.1047	Q9CPU2	NDUB2_MOUSE
Tmem30a	328	-0.1140	D3YVV1	D3YVV1_MOUSE
Hkdc1	915	-0.1441	Q91W97	HKDC1_MOUSE
Msn	577	-0.1451	P26041	MOES_MOUSE
Ndufa8	172	-0.1506	Q9DCJ5	NDUA8_MOUSE
Yif1b	251	-0.1514	D3YY42	D3YY42_MOUSE
Ace Dcp1	1312	-0.1657	P09470	ACE_MOUSE
Ncstn	708	-0.1674	P57716	NICA_MOUSE
Tmem43	400	-0.1814	Q9DBS1	TMM43_MOUSE
Mrpl3 mCG_14748	304	-0.1932	D3Z456	D3Z456_MOUSE
Ndrg2 Kiaa1248 Ndr2	371	-0.2066	Q9QYG0	NDRG2_MOUSE
Ndufs5	91	-0.2105	B1ARW4	B1ARW4_MOUSE
Mrpl2	304	-0.2132	B1B1D8	B1B1D8_MOUSE
Acat1	424	-0.2287	Q8QZT1	THIL_MOUSE
Myom2	1463	-0.2288	Q14BI5	Q14BI5_MOUSE
Pxmp2 mCG_134215	193	-0.2389	Q5D073	Q5D073_MOUSE
Slc44a1	656	-0.2408	A2AMH5	A2AMH5_MOUSE
Selenbp2	134	-0.2467	G3UZT2	G3UZT2_MOUSE
Nt5c2	586	-0.2480	E9Q9M1	E9Q9M1_MOUSE
Cox5b mCG_17741 mCG_2302(	129	-0.3013	Q9D881	Q9D881_MOUSE
Syngr1	234	-0.3132	O55100	SNG1_MOUSE
Anxa5 Anx5	319	-0.3230	P48036	ANXA5_MOUSE
Atp2a2	1044	-0.3400	O55143-2	AT2A2_MOUSE
Myh7	1935	-0.3455	Q91Z83	MYH7_MOUSE
Slc12a4	1087	-0.3455	F8WIJ0	F8WIJ0_MOUSE

Gnb1	273	-0.3519	H3BKR2	H3BKR2_MOUSE
Actg1 Actg	375	-0.3531	P63260	ACTG_MOUSE
Mtatp8 Atp8 mt-Atp8	67	-0.3594	P03930	ATP8_MOUSE
Lamp2 Lamp-2	415	-0.3640	P17047	LAMP2_MOUSE
Uqcrc1	480	-0.3713	Q9CZ13	QCR1_MOUSE
Mrpl37	423	-0.3745	Q921S7	RM37_MOUSE
Smpd2	419	-0.3746	O70572	NSMA_MOUSE
Anxa6	667	-0.3814	F8WIT2	F8WIT2_MOUSE
Rap1a mCG_10748	118	-0.3847	A0A0G2JDL9	A0A0G2JDL9_MOUSE
Cavin4 Murc	362	-0.3998	A2AMM0	CAVN4_MOUSE
Tmx1	124	-0.4453	F6V084	F6V084_MOUSE
d2 Cdgsh2 Noxp70 Zcd2	135	-0.4520	Q9CQB5	CISD2_MOUSE
Rpl19	194	-0.4779	A2A547	A2A547_MOUSE
Bsg	197	-0.4949	J3QP71	J3QP71_MOUSE
Cds2	194	-0.5383	F6S4G2	F6S4G2_MOUSE
Sspn mCG_15024	216	-0.5386	Q3TRE0	Q3TRE0_MOUSE
Lpgat1	409	-0.5590	E9QL80	E9QL80_MOUSE
Anxa2	176	-0.5837	B0V2N8	B0V2N8_MOUSE
at3 Grcc3f Mboat5 Oact5	487	-0.6015	Q91V01	MBOA5_MOUSE
Ap2m1 mCG_128452	433	-0.6270	Q3TWV4	Q3TWV4_MOUSE
Mrpl27	148	-0.6336	Q99N92	RM27_MOUSE
Uqcrq	82	-0.7405	Q9CQ69	QCR8_MOUSE
Rps21	83	-0.8363	Q9CQR2	RS21_MOUSE
Slc29a1	358	-0.8484	E9PXM6	E9PXM6_MOUSE
Cdipt	185	-0.9632	A0A0U1RP13	A0A0U1RP13_MOUSE
Tmem14c	114	-1.1246	Q9CQN6	TM14C_MOUSE
Glg1	1163	-1.2077	F8WHM5	F8WHM5_MOUSE
Cpne3 Kiaa0636	533	-1.2567	Q8BT60	CPNE3_MOUSE
Tmem205	173	-1.3642	A0A1L1SSA8	A0A1L1SSA8_MOUSE

Supplementary Data 2

ExoCarta TOP proteins

Gene Symbol
ACTG1
AHCY
ALB
ALDOA
ANXA11
ANXA2
ANXA4
ANXA5
ANXA6
ATP1A1
BSG
CCT2
CCT3
CD81
CLTC
ENO1
GNAI2
GNB1
GNB2
HSP90AB1
HSPA5
HSPA8
ITGB1
LAMP2
LDHA
LDHB
MSN
MVP
MYH9
PGK1
PKM
PRDX1
PRDX2
PTGFRN
RAB1A
RAC1
SLC16A1
STOM
TCP1
TFRC
TPI1
TUBA1C
VCP
YWHAE
YWHAZ
YWHAG
A2M

TUBA1B
RAC1
LGALS3BP
HSPA1A
GNAI2
ANXA1
RHOA
MFGE8
PRDX2
GDI2
EHD4
ACTN4
YWHAB
RAB7A
LDHB
GNAS
RAB5C
ARF1
ANXA6
ANXA11
ACTG1
KPNB1
EZR
ANXA4
ACLY
TUBA1C
TFRC
RAB14
HIST2H4A
GNB1
THBS1
RAN
RAB5A
PTGFRN
CCT5
CCT3
AHCY
UBA1
RAB5B
RAB1A
LAMP2
ITGA6
HIST1H4B
BSG
YWHAH
TUBA1A
TKT
TCP1
STOM
SLC16A1

RAB8A
MYH9
MVP

Supplementary Data 3

Antibody list

Name	Manufacturer	Species	Dilution
anti-CRYAB	abcam	mouse	1:1000 (WB)/ 1:100 IF
anti-GAPDH	Proteintech	mouse	1:50000 (WB)
anti-CD81	abcam	rabbit	1:1000 (WB)
anti-TSG101	abcam	rabbit	1:1000 (WB)
anti-GM130	abcam	rabbit	1:1000 (WB)
anti-KI67	abcam	rabbit	1:200 (IF)
Anti-LAMP1	Sigma Aldrich	rabbit	1:200 (IF)
Anti-CTNNB1	BD Transduction	mouse	1:200 (IF)
Anti-TUBA1B	Proteintech	mouse	1:200 (IF)
Anti-GAPDH	Cell signaling technology	mouse	1:1000 (WB)
Anti-Calnexin	abcam	rabbit	1:1000 (WB)
Anti-Vinculin	Cell signaling technology	rabbit	1:1000 (WB)
Anti-Ubiquitin (P4D1)	Cell signaling technology	mouse	1:1000
Phospho-β-Catenin (Ser675)	Cell signaling technology	rabbit	1:200 (IF)/1:1000 (WB)
Non-phospho (Active) β-Catenin (Ser33/37/Thr41) Antibody	Cell signaling technology	rabbit	1:1000 (WB)
Anti-rabbit IgG, HRP-linked Antibody	Cell signaling technology	rabbit	1:10000 (WB)
Anti-mouse IgG, HRP-linked Antibody	Cell signaling technology	mouse	1:10000 (WB)
