# Supplementary material for: Single-cell transcriptomics reveal extracellular vesicles secretion with a cardiomyocyte proteostasis signature during pathological remodeling
Source: Commun Biol. 2023 Jan 21;6:79. doi: 10.1038/s42003-022-04402-9 (PMC9867722; doi:10.1038/s42003-022-04402-9)
Supplement: Supplementary file 5 — Reporting Summary [file 42003_2022_4402_MOESM5_ESM.pdf]

Corresponding author(s): Laura C. Zelarayán

Last updated by author(s): Dec 21, 2022

Reporting Summary

Nature Portfolio wishes to improve the reproducibility of the work that we publish. This form provides structure for consistency and transparency in reporting. For further information on Nature Portfolio policies, see our Editorial Policies and the Editorial Policy Checklist.

Statistics

For all statistical analyses, confirm that the following items are present in the figure legend, table legend, main text, or Methods section.

n/a	Confirmed	
☐	☒	The exact sample size (*n*) for each experimental group/condition, given as a discrete number and unit of measurement
☐	☒	A statement on whether measurements were taken from distinct samples or whether the same sample was measured repeatedly
☐	☒	The statistical test(s) used AND whether they are one- or two-sided<br>*Only common tests should be described solely by name; describe more complex techniques in the Methods section.*
☒	☐	A description of all covariates tested
☒	☐	A description of any assumptions or corrections, such as tests of normality and adjustment for multiple comparisons
☐	☒	A full description of the statistical parameters including central tendency (e.g. means) or other basic estimates (e.g. regression coefficient) AND variation (e.g. standard deviation) or associated estimates of uncertainty (e.g. confidence intervals)
☐	☒	For null hypothesis testing, the test statistic (e.g. *F*, *t*, *r*) with confidence intervals, effect sizes, degrees of freedom and *P* value noted<br>*Give P values as exact values whenever suitable.*
☒	☐	For Bayesian analysis, information on the choice of priors and Markov chain Monte Carlo settings
☒	☐	For hierarchical and complex designs, identification of the appropriate level for tests and full reporting of outcomes
☐	☒	Estimates of effect sizes (e.g. Cohen's *d*, Pearson's *r*), indicating how they were calculated

Our web collection on statistics for biologists contains articles on many of the points above.

Software and code

Policy information about availability of computer code

Data collection	For proteomics, protein identification was achieved using ProteinPilot Software version 5.0 build 4769 (AB Sciex) at “thorough” settings. The combined qualitative analyses were searched against the UniProtKB mouse reference proteome (revision 12-2017, 60717 entries) augmented with a set of 52 known common laboratory contaminants to identify proteins at a False Discovery Rate (FDR) of 1%.<br>For single cell library preparation, heart cells were singularized and were distributed on 5,184 nanowell chips ICELL8 250v Chip (ICELL8 System, Takara Bio).
Data analysis	For proteomics, spectral library generation and SWATH peak extraction were achieved in PeakView Software version 2.1 build 11041 (AB Sciex) using the SWATH quantitation microApp version 2.0 build 2003. Following retention time correction using the iRT standard, peak areas were extracted using information from the MS/MS library at an FDR of 1%76. The resulting peak areas were then summed to peptide and finally protein area values per injection, which were used for further statistical analysis in Perseus 1.5.6.0 software (Max Planck Institute for Biochemistry, Martinsried, Germany). STRING (Search Tool for the Retrieval of Interacting Genes/Proteins) (http://string-db.org/) was used for network analysis.<br>Raw sequencing files (bcl-files) were converted into a single fastq file using Illumina bcl2fastq software (v2.20.0.422) for each platform. Each fastq file was demultiplexed and analysed using the Cogent NGS analysis pipeline (CogentAP) from Takara Bio (v1.0). In brief, cogent demux wrapper function was used to allocate the reads to the cells based on the cell barcodes provided in the well-list files. Subsequently, cogent analyze wrapper function performed read trimming with cutadapt(Martin 2011)(version 3.2), genome alignment to Mus musculus genome GRCm38 using STAR(Dobin, Davis et al. 2013) (version 2.7.7a), read counting for exonic, genomic and mitochondrial regions using featureCounts(Liao, Smyth et al. 2013) (version 2.0.1) and utilizing Mus musculus gene annotation version 102 from ENSEMBL and generating a gene matrix with number of reads expressed for each cell in each gene. Raw gene matrices underwent quality control (QC) filtering for cells and genes using the following parameters: (a) for cells, only those with at least 2500 genes and less than 30 % of mitochondrial reads, and (b) for genes, only those containing at least 100 reads mapped to them from at least 3 different cells. The bioinformatic analysis was performed

using the Seurat package (version 4.1.2)(Hao, Hao et al. 2021). Expression matrices were splited by Chip and, after normalization by 'NormalizeData' using default settings and selection of the 2000 most variable features using 'FidVariableFeatures'. Integration of the datasets was performed by the standard parameters of 'IntegrateData'. Data was scaled and centered by 'ScaleData', regressing out the variability caused by the mitochondrial percentage and the Depth of the sequencing. Dimensions were reduced using 'RunPCA'. Afterwards, 'FindNeightbors' (dimension parameter = 1:9) and 'FindClusters' (granularity parameter = 0.3) were run and the clusters were visualized, after UMAP reduction was calculated by using Louvian algorithm. Gene ontology (GO) analyses were performed using default parameters and stringency in 'ClueGO' (Cytoscape)(Bindea, Mlecnik et al. 2009) and DAVIS. The significant gene ontologies (GO) are shown with p < 0.05. The cardiomyocyte stress, hypoxia and EV scores were calculated by the expression of established markers per cell(Nicin, Schroeter et al. 2022). Ggplot2(Wickham 2016), Dplyr(Wickham H 2022) and EnhancedVolcano packages were used for data visualization(Blighe 2018).

For manuscripts utilizing custom algorithms or software that are central to the research but not yet described in published literature, software must be made available to editors and reviewers. We strongly encourage code deposition in a community repository (e.g. GitHub). See the Nature Portfolio guidelines for submitting code & software for further information.

Data

Policy information about availability of data

All manuscripts must include a data availability statement. This statement should provide the following information, where applicable:

- Accession codes, unique identifiers, or web links for publicly available datasets
- A description of any restrictions on data availability
- For clinical datasets or third party data, please ensure that the statement adheres to our policy

The mass spectrometry proteomic dataset generated during the current study is available in the ProteomeXchange Consortium via the PRIDE(Perez-Riverol, Csordas et al. 2018) partner repository with the dataset identifier PXD031113, the bulk RNAseq data is available at NCBI GEO under accession GSE97763, the raw and normalized Single Cell RNAseq data is available at https://owncloud.¬gwdg.de/index.php/s/NeSJGuf2o2yc2qn. Reporting of the EV protocol is available at evtrack.org (IDEV220418) (Van Deun, Mestdagh et al. 2017) including the MISEV2018(Théry, Witwer et al. 2018). The uncropped Western blots in Supplementary Figure 10.

Human research participants

Policy information about studies involving human research participants and Sex and Gender in Research.

Reporting on sex and gender	na
Population characteristics	na
Recruitment	na
Ethics oversight	na

Note that full information on the approval of the study protocol must also be provided in the manuscript.

Field-specific reporting

Please select the one below that is the best fit for your research. If you are not sure, read the appropriate sections before making your selection.

☒ Life sciences ☐ Behavioural & social sciences ☐ Ecological, evolutionary & environmental sciences

For a reference copy of the document with all sections, see nature.com/documents/nr-reporting-summary-flat.pdf

Life sciences study design

All studies must disclose on these points even when the disclosure is negative.

Sample size	For single cell analysis, a priori: Compute required sample size, was used. Sample size per group=105 (Effect size d=0.5, power (1-β err prob)=0.95, comparison two independent groups). For mice interventions (TAC), sample size per group=3 (Effect size d=0.9, power (1-β err prob)=0.85, comparison two independent groups).
Data exclusions	na
Replication	Biological replicates were used for SCS. Biological and technical replicates were used for proteomics and in vitro studies.
Randomization	na
Blinding	Investigators were blinded for proteomics, transcriptomics, functional and Western blot experiments.

Behavioural & social sciences study design

All studies must disclose on these points even when the disclosure is negative.

Study description	*Briefly describe the study type including whether data are quantitative, qualitative, or mixed-methods (e.g. qualitative cross-sectional, quantitative experimental, mixed-methods case study).*
Research sample	*State the research sample (e.g. Harvard university undergraduates, villagers in rural India) and provide relevant demographic information (e.g. age, sex) and indicate whether the sample is representative. Provide a rationale for the study sample chosen. For studies involving existing datasets, please describe the dataset and source.*
Sampling strategy	*Describe the sampling procedure (e.g. random, snowball, stratified, convenience). Describe the statistical methods that were used to predetermine sample size OR if no sample-size calculation was performed, describe how sample sizes were chosen and provide a rationale for why these sample sizes are sufficient. For qualitative data, please indicate whether data saturation was considered, and what criteria were used to decide that no further sampling was needed.*
Data collection	*Provide details about the data collection procedure, including the instruments or devices used to record the data (e.g. pen and paper, computer, eye tracker, video or audio equipment) whether anyone was present besides the participant(s) and the researcher, and whether the researcher was blind to experimental condition and/or the study hypothesis during data collection.*
Timing	*Indicate the start and stop dates of data collection. If there is a gap between collection periods, state the dates for each sample cohort.*
Data exclusions	*If no data were excluded from the analyses, state so OR if data were excluded, provide the exact number of exclusions and the rationale behind them, indicating whether exclusion criteria were pre-established.*
Non-participation	*State how many participants dropped out/declined participation and the reason(s) given OR provide response rate OR state that no participants dropped out/declined participation.*
Randomization	*If participants were not allocated into experimental groups, state so OR describe how participants were allocated to groups, and if allocation was not random, describe how covariates were controlled.*

Ecological, evolutionary & environmental sciences study design

All studies must disclose on these points even when the disclosure is negative.

Study description	*Briefly describe the study. For quantitative data include treatment factors and interactions, design structure (e.g. factorial, nested, hierarchical), nature and number of experimental units and replicates.*
Research sample	*Describe the research sample (e.g. a group of tagged Passer domesticus, all Stenocereus thurberi within Organ Pipe Cactus National Monument), and provide a rationale for the sample choice. When relevant, describe the organism taxa, source, sex, age range and any manipulations. State what population the sample is meant to represent when applicable. For studies involving existing datasets, describe the data and its source.*
Sampling strategy	*Note the sampling procedure. Describe the statistical methods that were used to predetermine sample size OR if no sample-size calculation was performed, describe how sample sizes were chosen and provide a rationale for why these sample sizes are sufficient.*
Data collection	*Describe the data collection procedure, including who recorded the data and how.*
Timing and spatial scale	*Indicate the start and stop dates of data collection, noting the frequency and periodicity of sampling and providing a rationale for these choices. If there is a gap between collection periods, state the dates for each sample cohort. Specify the spatial scale from which the data are taken*
Data exclusions	*If no data were excluded from the analyses, state so OR if data were excluded, describe the exclusions and the rationale behind them, indicating whether exclusion criteria were pre-established.*
Reproducibility	*Describe the measures taken to verify the reproducibility of experimental findings. For each experiment, note whether any attempts to repeat the experiment failed OR state that all attempts to repeat the experiment were successful.*
Randomization	*Describe how samples/organisms/participants were allocated into groups. If allocation was not random, describe how covariates were controlled. If this is not relevant to your study, explain why.*
Blinding	*Describe the extent of blinding used during data acquisition and analysis. If blinding was not possible, describe why OR explain why blinding was not relevant to your study.*

Did the study involve field work? ☐ Yes ☐ No

Field work, collection and transport

Field conditions

Describe the study conditions for field work, providing relevant parameters (e.g. temperature, rainfall).

Location

State the location of the sampling or experiment, providing relevant parameters (e.g. latitude and longitude, elevation, water depth).

Access & import/export

Describe the efforts you have made to access habitats and to collect and import/export your samples in a responsible manner and in compliance with local, national and international laws, noting any permits that were obtained (give the name of the issuing authority, the date of issue, and any identifying information).

Disturbance

Describe any disturbance caused by the study and how it was minimized.

Reporting for specific materials, systems and methods

We require information from authors about some types of materials, experimental systems and methods used in many studies. Here, indicate whether each material, system or method listed is relevant to your study. If you are not sure if a list item applies to your research, read the appropriate section before selecting a response.

Materials & experimental systems

n/a	Involved in the study
☐	☒ Antibodies
☐	☒ Eukaryotic cell lines
☐	☐ Palaeontology and archaeology
☐	☒ Animals and other organisms
☐	☐ Clinical data
☐	☐ Dual use research of concern

Methods

n/a	Involved in the study
☐	☐ ChIP-seq
☐	☐ Flow cytometry
☐	☐ MRI-based neuroimaging

Antibodies

Antibodies used

Name	Manufacturer	Species	Dilution
anti-CRYAB	abcam	mouse	1:1000 (WB)/ 1:100 IF
anti-GAPDH	Proteintech	mouse	1:50000 (WB)
anti-CD81	abcam	rabbit	1:1000 (WB)
anti-TSG101	abcam	rabbit	1:1000 (WB)
anti-GM130	abcam	rabbit	1:1000 (WB)
anti-KI67	abcam	rabbit	1:200 (IF)
Anti-LAMP1	Sigma Aldrich	rabbit	1:200 (IF)
Anti-CTNNB1	BD Transduction	mouse	1:200 (IF)
Anti-TUBA1B	Proteintech	mouse	1:200 (IF)
Anti-GAPDH	Cell signaling technology	mouse	1:1000 (WB)
Anti-Calnexin	abcam	rabbit	1:1000 (WB)
Anti-Vinculin	Cell signaling technology	rabbit	1:1000 (WB)
Anti-Ubiquitin (P4D1)	Cell signaling technology	mouse	1:1000
Phospho-β-Catenin (Ser675)	Cell signaling technology	rabbit	1:200 (IF)
Non-phospho (Active) β-Catenin (Ser33/37/Thr41) Antibody	Cell signaling technology	rabbit	1:1000 (WB)
Anti-rabbit IgG, HRP-linked Antibody	Cell signaling technology	rabbit	1:10000 (WB)
Anti-mouse IgG, HRP-linked Antibody	Cell signaling technology	mouse	1:10000 (WB)

Validation

see above in the table (antibody used)

Eukaryotic cell lines

Policy information about cell lines and Sex and Gender in Research

Cell line source(s)

Induced pluripotent stem cells (TC1133); Neuro2a cells (N2A) (CCL-131)

Authentication

TC1133 / RUCDRi002-A

Mycoplasma contamination

confirmed negative for mycoplasma

Commonly misidentified lines
(See ICLAC register)

TC1133 or RUCDRi002-A

Palaeontology and Archaeology

Specimen provenance	na
Specimen deposition	na
Dating methods	na

☐ Tick this box to confirm that the raw and calibrated dates are available in the paper or in Supplementary Information.

Ethics oversight	na

Note that full information on the approval of the study protocol must also be provided in the manuscript.

Animals and other research organisms

Policy information about studies involving animals; ARRIVE guidelines recommended for reporting animal research, and Sex and Gender in Research

Laboratory animals	mice (mus musculus), male and females, 17,5 months-old (average)
Wild animals	na
Reporting on sex	na
Field-collected samples	na
Ethics oversight	All animal experiments were approved by the Lower Saxony (AZ-G 20.3434) animal review board (LAVES).

Note that full information on the approval of the study protocol must also be provided in the manuscript.

Clinical data

Policy information about clinical studies

All manuscripts should comply with the ICMJE guidelines for publication of clinical research and a completed CONSORT checklist must be included with all submissions.

Clinical trial registration	na
Study protocol	na
Data collection	na
Outcomes	na

Dual use research of concern

Policy information about dual use research of concern

Hazards

Could the accidental, deliberate or reckless misuse of agents or technologies generated in the work, or the application of information presented in the manuscript, pose a threat to:

No	Yes	
☒	☐	Public health
☒	☐	National security
☒	☐	Crops and/or livestock
☒	☐	Ecosystems
☒	☐	Any other significant area

Experiments of concern

Does the work involve any of these experiments of concern:

No	Yes	
☒	☐	Demonstrate how to render a vaccine ineffective
☒	☐	Confer resistance to therapeutically useful antibiotics or antiviral agents
☒	☐	Enhance the virulence of a pathogen or render a nonpathogen virulent
☒	☐	Increase transmissibility of a pathogen
☒	☐	Alter the host range of a pathogen
☒	☐	Enable evasion of diagnostic/detection modalities
☒	☐	Enable the weaponization of a biological agent or toxin
☒	☐	Any other potentially harmful combination of experiments and agents

ChIP-seq

Data deposition

☐ Confirm that both raw and final processed data have been deposited in a public database such as GEO.

☐ Confirm that you have deposited or provided access to graph files (e.g. BED files) for the called peaks.

Data access links<br>*May remain private before publication.*	na
Files in database submission	na
Genome browser session<br>(e.g. UCSC)	na

Methodology

Replicates	na
Sequencing depth	na
Antibodies	na
Peak calling parameters	na
Data quality	na
Software	na

Flow Cytometry

Plots

Confirm that:

☐ The axis labels state the marker and fluorochrome used (e.g. CD4-FITC).

☐ The axis scales are clearly visible. Include numbers along axes only for bottom left plot of group (a 'group' is an analysis of identical markers).

☐ All plots are contour plots with outliers or pseudocolor plots.

☐ A numerical value for number of cells or percentage (with statistics) is provided.

Methodology

Sample preparation	na
Instrument	na
Software	na
Cell population abundance	na

Gating strategy | na

☐ Tick this box to confirm that a figure exemplifying the gating strategy is provided in the Supplementary Information.

Magnetic resonance imaging

Experimental design

Design type | na

Design specifications | na

Behavioral performance measures | na

Acquisition

Imaging type(s) | na

Field strength | na

Sequence & imaging parameters | na

Area of acquisition | na

Diffusion MRI ☐ Used ☐ Not used

Preprocessing

Preprocessing software | na

Normalization | na

Normalization template | na

Noise and artifact removal | na

Volume censoring | na

Statistical modeling & inference

Model type and settings | na

Effect(s) tested | *Define precise effect in terms of the task or stimulus conditions instead of psychological concepts and indicate whether ANOVA or factorial designs were used.*

Specify type of analysis: ☐ Whole brain ☐ ROI-based ☐ Both

Statistic type for inference (See Eklund et al. 2016) | na

Correction | na

Models & analysis

n/a	Involved in the study
☐	☐ Functional and/or effective connectivity
☐	☐ Graph analysis
☐	☐ Multivariate modeling or predictive analysis

Functional and/or effective connectivity | *Report the measures of dependence used and the model details (e.g. Pearson correlation, partial correlation, mutual information).*

Graph analysis | *Report the dependent variable and connectivity measure, specifying weighted graph or binarized graph, subject- or group-level, and the global and/or node summaries used (e.g. clustering coefficient, efficiency, etc.).*

Multivariate modeling and predictive analysis | *Specify independent variables, features extraction and dimension reduction, model, training and evaluation metrics.*
